# Toward Sustainable Lithium‐Ion Batteries: Recycling and Reuse Strategies for Spent Graphite Anodes

**DOI:** 10.1002/smll.202509952

**Published:** 2025-12-17

**Authors:** Zhifei Mao, Jingshan Chai, Ruigang Wang

**Affiliations:** ^1^ Department of Chemical Engineering and Materials Science Michigan State University East Lansing MI 48824 USA

**Keywords:** graphite anode, hydrometallurgy, Lithium‐ion batteries, pyrometallurgy, recycling and reuse

## Abstract

The growing demand for lithium‐ion batteries (LIBs) has intensified the need for sustainable graphite recycling and reuse. Spent graphite anodes, accounting for a significant portion of battery waste, suffer from structural degradation, surface contamination, and the formation of complex impurities, posing challenges for direct reuse. This review introduces the general degradation origins of spent graphite anodes and focuses on recycling, regeneration, and modification strategies, including hydrometallurgical and pyrometallurgical approaches. The emerging technique of Flash Joule heating (FJH) is also discussed. Regenerated graphite generally fails to meet the desired electrochemical performance and therefore needs further modification. Strategies including surface engineering, structural regulation, and hybridization with other materials have been widely explored, leading to improved structural stability, fast‐charging capability, and even higher energy density. Finally, the key challenges hindering large‐scale application are identified. This review aims to provide comprehensive insights into sustainable graphite recycling and reuse, promoting the development of high‐performance and environmentally responsible LIBs.

## Graphical abstract

This review outlines the degradation mechanisms of spent graphite anodes in lithium‐ion batteries and highlights key recycling, regeneration, and modification strategies, spanning hydrometallurgical and pyrometallurgical methods. It also examines advances in surface engineering, structural tuning, and material hybridization, offering insights that support sustainable graphite reuse and the development of high‐performance, environmentally responsible lithium‐ion batteries.

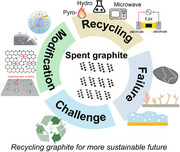

## 1 Introduction

**Figure 1:**
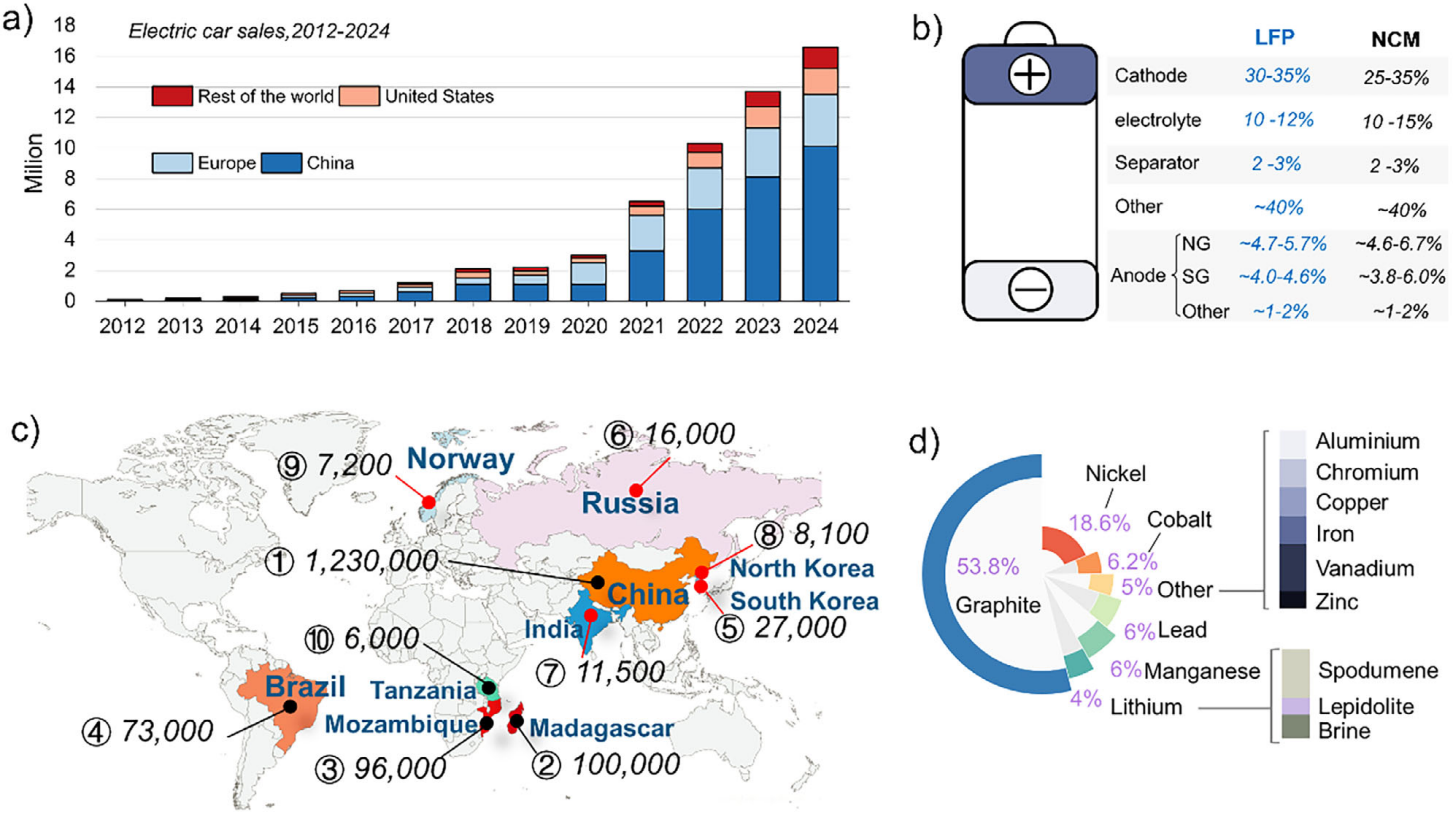
(a) Trends in global electric vehicle sales (Source: IEA. Global EV Outlook 2024). (b) Breakdown of the mass proportion of each component in LFP and NCM cells. (c) The world's leading graphite producer countries and their natural graphite minerals (in tons). (d) Forecasted battery mineral demand to 2050 (Source: World Bank Group, May 2020).

Batteries store and deliver electrical energy that powers a wide range of modern technologies, including portable electronics and renewable energy systems. Lithium‐ion batteries (LIBs) are widely employed in electric vehicles (EVs) because of their high energy density and compact size. EVs sales reached 16.6 million in 2024, with ≈91.6% occurring in China, the United States and Europe (**Figure** **1**a). This represents more than 5.5‐fold increase compared to 2020, just 4 years earlier. While EV batteries have a limited lifespan (10–20 years), the industry is not yet prepared to manage the resulting large volume of spent batteries. Direct landfilling would result in enormous resource waste, pose environmental threats, and present hazardous safety risks. Therefore, researchers are highly motivated to develop cost‐effective and sustainable battery recycling technologies. In 2024, the U.S. government announced $63 million in funding to advance battery recycling [Source: *ESS News*, August 2024]. Using materials from used batteries to produce new ones is a win‐win approach: it ensures a stable supply of raw materials, reduces environmental threats, and enhances cost‐effectiveness.

In commercial lithium iron phosphate (LiFePO_4_, LFP) and ternary nickel‐cobalt‐manganese (NCM) LIBs, the anode (approximately 98% is graphite) and cathode together account for approximately half of the battery's total mass (Figure 1b). Graphite is the anode material of choice in modern state‐of‐the‐art LIBs, accounting for ≈95% of the anode market, with the remaining 5% made up of silicon and other materials. China is the world's leading producer of natural graphite (NG), contributing an estimated 90% of total global production (Figure 1c, Source: NAI500 News, August 2024).

In both the United States and the European Union, where graphite reserves and production are limited, NG is classified as a critical mineral (Source: 2021 Minerals Yearbook).^[^ ^1^, ^2^ ^]^ The average EVs battery contains 50–100 kg of graphite, which is the highest percentage of minerals in the battery (Source: The key minerals in an EV battery, Mining Industry). By 2050, graphite is expected to be the most in‐demand battery mineral, significantly surpassing other materials (Figure 1d) (Source: World Bank Group, May 2020).

**Figure 2:**
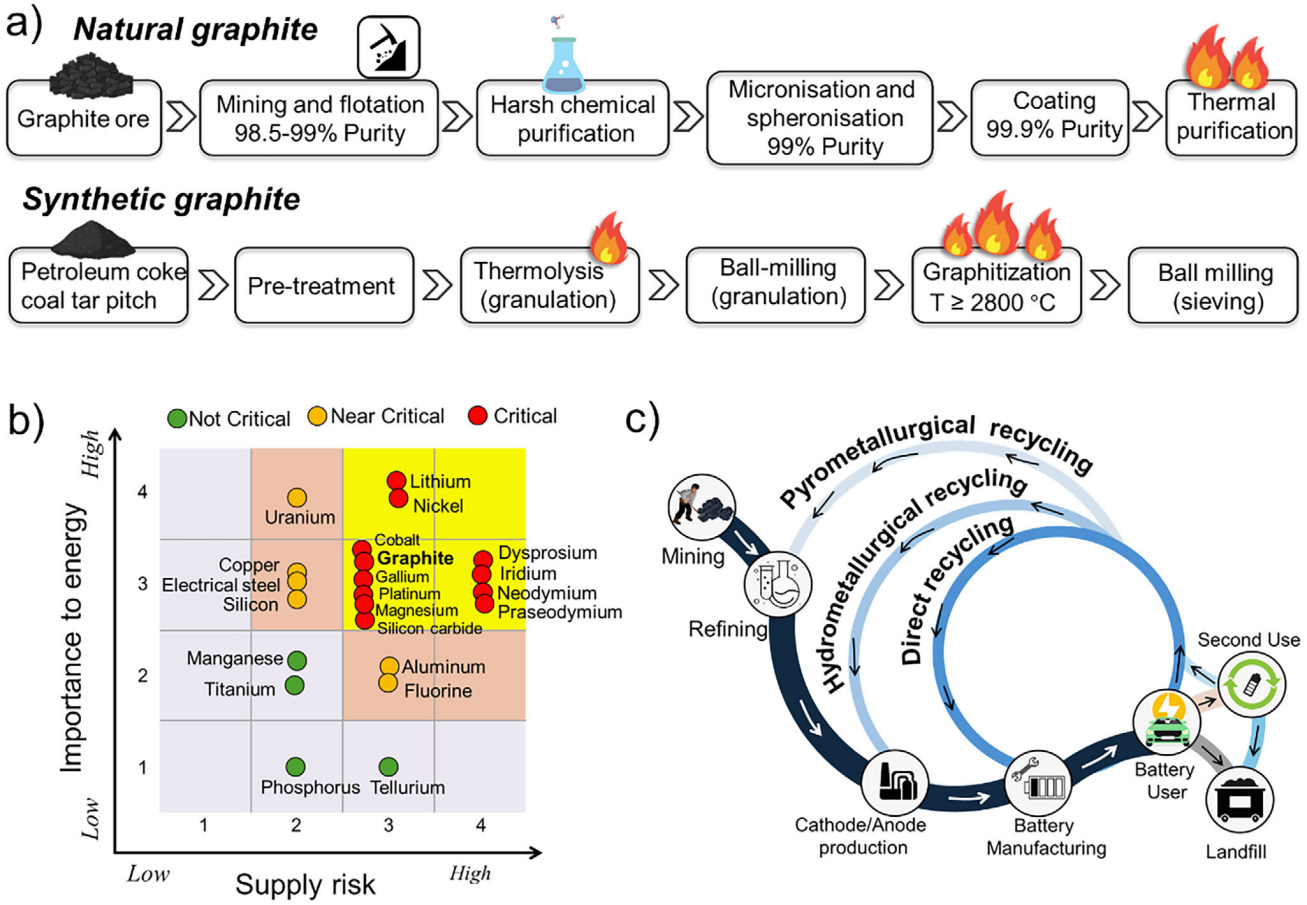
(a) Production process of natural and synthetic graphite. (b) Medium‐term (2025–2035) criticality matrix (Source: DOE Draft Critical Materials Assessment Report. https://www.energy.gov/eere/ammto/articles/2023‐doe‐critical‐materials‐assessment). (c) Schematic of the LIBs life cycle and recycling pathways.

Graphite can be distinguished between artificial or synthetic graphite (SG) and NG. The production of graphite mainly follows two different routes (**Figure** **2**a). NG is formed from organic matter that has been subjected to high temperatures and pressures over an extended period of time. Production of NG starts with mining and flotation, combined with the successive chemical purification, micronisation and spheronisation, coating and thermal purification. To achieve purity levels ≥99.0% (battery grade purity >99.9%), chemical treatment and thermal purification treatments (≥2000 °C) are necessary.^[^ ^3^, ^4^ ^]^ SG is typically prepared from precursor petroleum coke (often mixed with coal‐tar pitch) using a high‐temperature graphitization treatment (≥2800 °C). The graphitization is a high energy consumption and high cost process (≈10–12 kWh kg^−1^),^[^ ^5^ ^]^ and the costs and greenhouse gas emissions of SG are higher than those of NG. Graphitization of SG results in emissions of 13.8 kgCO_2_‐eq/kg and an energy consumption of 45.9 MJ kg^−1^.^[^ ^6^ ^]^ The production cost of graphite varies significantly with its source: NG typically costs around US $2000–3000 t^−1^, while SG ranges from US $7000–10000 t^−1^ due to high‐temperature graphitization. To achieve more sustainable LIBs and reduce their carbon footprint, it is essential to recycle graphite from used LIBs and recycled graphite is estimated at US $1500–2500 t^−1^. The recycled waste graphite retains its ordered structure and does not require high‐temperature reprocessing, hence reducing costs.^[^ ^7^, ^8^ ^]^

There are six critical materials identified due to their high importance to energy systems and elevated supply risk in the medium term (2025–2035). These include cobalt, graphite, gallium, platinum, magnesium, and silicon carbide (Figure 2b). Graphite is indispensable for LIBs, serving as the dominant anode material due to its unique combination of low cost, abundance, high energy and power densities, and long cycle life.^[^ ^9^ ^]^ The growing demand for EVs and energy storage systems has significantly intensified the need for graphite, putting additional strain on its supply chain. As a result, graphite's criticality highlights both its pivotal role in advancing clean energy technologies and the pressing challenges associated with its resource security and market availability. Given the critical role and increasing scarcity of graphite and other key battery component materials, it is essential to develop sustainable strategies to mitigate supply risks and ensure long‐term resource security. One of the most effective approaches is the recycling of spent LIBs, which enables the recovery and reuse of valuable materials, such as graphite, cobalt, and nickel. The battery life cycle begins with mining and refining raw materials, followed by battery component production and battery manufacturing (Figure 2c). After their use in EVs or other applications, batteries may be sent to landfills, repurposed for second‐life applications, or processed through various recycling methods. The three primary recycling routes are pyrometallurgical, hydrometallurgical, and direct recycling.

Previous reviews have discussed the environmental and economic importance of graphite recycling and provided overviews of existing recovery technologies.^[^ ^10^, ^11^ ^]^ However, most of these works focus on the general rationale and process classifications, with limited discussion of structural regeneration, surface modification, and electrochemical reintegration of recycled graphite. In this review, the degradation mechanisms, recycling strategies, and modification approaches of spent graphite are systematically analyzed, emphasizing the correlation between degradation, regeneration, and electrochemical performance. Recent progress in hydrometallurgical, pyrometallurgical, and hybrid recycling technologies, as well as surface and structural engineering strategies, is summarized to promote greener and more sustainable development of LIBs.

## 2 Degradation Mechanisms of Graphite Anode and Its Recycling Processes

### 2.1 Degradation Mechanisms

**Figure 3:**
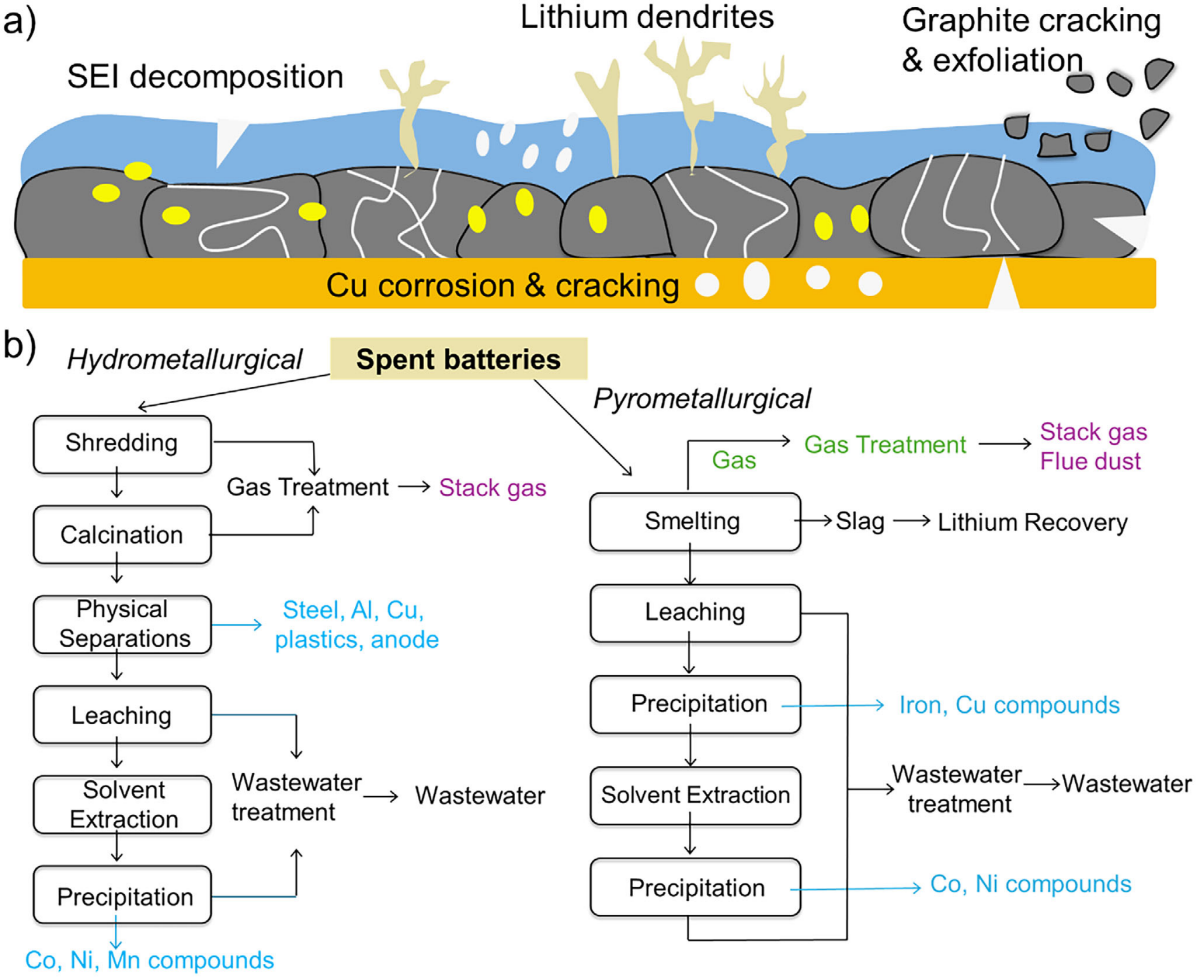
(a) Schematic of the failure mechanisms of spent graphite anode. (b) A generic pyrometallurgical and hydrometallurgical recycling process. Data adapted from the EverBatt: A Closed‐loop Battery Recycling Cost and Environmental Impacts Model (Argonne National Laboratory, 2019).

Graphite degradation in LIBs is primarily driven by several failure mechanisms, including solid electrolyte interphase (SEI) layer breakdown,^[^ ^12^, ^13^ ^]^ lithium dendrite growth,^[^ ^14^, ^15^, ^16^ ^]^ graphite particle fracture,^[^ ^17^, ^18^, ^19^ ^]^ and copper current‐collector corrosion^[^ ^20^, ^21^, ^22^ ^]^ (**Figure** **3**a). Instability of the SEI layer increases interfacial resistance and leads to continuous electrolyte decomposition, which consumes Li^+^ ions and reduces battery efficiency. Lithium dendrites can grow and puncture the separator, potentially causing internal short circuits and posing safety hazards. Graphite particles undergo mechanical stress from repeated Li^+^ intercalation and deintercalation, leading to cracks, volume expansion, and exfoliation. Additionally, copper current collectors can corrode and dissolve, causing contamination and further electrode degradation. Together, these failure mechanisms contribute to the performance decline and the eventual failure of graphite anodes in LIBs. The surface of waste graphite is often extensively coated with deposits from electrolyte decomposition.^[^ ^8^ ^]^ Additionally, contamination from other battery components can become mixed with the graphite anode, introducing various impurities. To effectively reuse waste graphite as an anode material in LIBs, it is essential to thoroughly remove these contaminants and repair any structural damage.

### 2.2 Separation and Recycling of Graphite Anode

Graphite recovery from spent LIBs involves a series of pretreatment steps to enable efficient separation and impurity removal. The process typically begins with discharging, which ensures the safe handling of spent LIBs prior to further processing. Common discharge methods include chemical discharge using salt such as NaCl,^[^ ^23^ ^]^ alkaline or acid solutions,^[^ ^24^ ^]^ cryogenic freezing with liquid nitrogen,^[^ ^25^ ^]^ and solid conductor discharge utilizing conductive materials like copper powder or graphite.^[^ ^26^ ^]^ After discharging, spent LIBs undergo mechanical dismantling processes, such as crushing, sieving, and physical separation, to liberate the electrode materials.^[^ ^27^ ^]^ Graphite is subsequently separated from the copper current collector through techniques such as high‐temperature pyrolysis or solvent dissolution, followed by purification steps including acid leaching and thermal treatment to remove impurities such as metals, binders, and residual electrolytes.^[^ ^28^ ^]^ The recovered graphite can be directly regenerated or further modified to enhance its electrochemical properties for reuse in battery or other applications.

Currently, the global recycling rate of LIBs is only 3%, with most efforts focused on recovering valuable metals, such as Li, Co, Ni, and Mn.^[^ ^29^, ^30^, ^31^ ^]^ Recycling begins with the separation of materials, and commercial approaches typically rely on two primary methods: pyrometallurgy and hydrometallurgy (Figure 3b and **Table** **1**). Pyrometallurgy involves dismantling and crushing spent batteries, followed by heating above 500 °C to remove the electrolyte. Further heating above 1400 °C melts metals like cobalt, nickel, copper, and iron for recovery and purification, while other metal oxides decompose into useful compounds. In contrast, hydrometallurgy operates at much lower temperatures, typically below 100 °C, by dissolving lithium‐ion cells in acids, primarily sulfuric acids. This process extracts metals, including lithium, from the cathode. However, it requires large volumes of solvents and generates significant waste and pollutants.

**Table 1:** Fates of various components of batteries in industrial recycling methods.

	Pyrometallurgical	Hydrometallurgical	Direct	Performance
Active cathode materials	Recycle	Recycle	Recycle	Direct route retains structure; others need re‐synthesis.
Graphite	Burn for energy	Recycle	Recycle	Direct recycling preserves crystallinity and reversibility.
Copper	Recycle	Recycle	Recycle	High recovery efficiency in all routes.
Aluminum	Landfill	Recycle	Recycle	Hydro/direct routes recover Al efficiently.
Steel	Recycle	Recycle	Recycle	Comparable recovery efficiency.
Electrolyte	Burn for energy	Burn for energy	Recycle	Hydro/direct allow Li salt and solvent recovery.
Carbon black	Burn for energy	Landfill	Recycle	Direct route enables reuse as additive.
PVDF	Burn for energy	Landfill	Recycle	Direct recycling regenerates binder and cuts emissions.

Note: Data adapted from EverBatt: A Closed‐loop Battery Recycling Cost and Environmental Impacts Model (Argonne National Laboratory, 2019).

The recycling of spent graphite from LIBs typically follows a three‐step process involving mechanical disassembly,^[^ ^32^ ^]^ valuable metal recovery,^[^ ^33^ ^]^ and graphite regeneration.^[^ ^34^ ^]^ Initially, exhausted batteries are physically dismantled to separate current collectors and active material powders. Subsequently, extraction techniques are employed to recover high‐value metallic components such as cobalt, nickel, and manganese from the composite electrode powders. The remaining graphite‐rich fraction is then subjected to purification and regeneration processes aimed at removing binders, electrolyte residues, and inorganic impurities. However, residual impurities and structural damage often limit the direct reuse of recycled graphite (R‐Gr), leading to reduced electrochemical performance and shorter battery life. Therefore, eliminating impurities and restoring the structural integrity of R‐Gr are essential for recovering its electrochemical functionality.

## 3 Recycling of Spent Graphite

Recycling spent graphite for use as an electrode material requires the effective removal of impurities (**Table** **2**) and the restoration of its structural integrity.^[^ ^36^ ^]^ The recycling of spent LIBs primarily relies on pyrometallurgical and hydrometallurgical processes (Figure 3b and Table 1). However, a major drawback of traditional pyrometallurgical recycling is its inability to recover lithium, as the process primarily focuses on extracting other valuable metals while burning off organic electrolytes and binders. In contrast, hydrometallurgical methods involve dissolving dismantled electrodes in concentrated acids, producing metal‐rich leach solutions. These solutions are then processed using various techniques to selectively recover Individual metals, including lithium, cobalt, nickel, and manganese, making hydrometallurgical recycling a more effective approach for lithium recovery. The lithium in spent graphite mainly originates from the SEI (composed of compounds such as Li_2_CO_3_, LiF, Li_2_O, ROCO_2_Li, ROLi, and (ROCO_2_Li)_2_) on the graphite surface. Additionally, some LiPF_6_ residues are present. While some components are water‐soluble, others are insoluble. Residues such as metals, lithium compounds and electrolytes can be removed from waste graphite via leaching methods, and the commonly used leaching agents are acids.

**Table 2:** Metal impurity of spent graphite.^[^ ^35^ ^]^

Metal element	Li	Cu	Na	Al	Co	Mn
Content (g kg^−1^)	35.6330	5.2134	1.3221	0.2400	0.2156	0.2113

### 3.1 Industrial Recycling Strategies

In recent years, industrial efforts toward graphite recovery have accelerated worldwide, reflecting its growing importance in achieving closed‐loop battery manufacturing. In China, Eastcarb has launched the nation's first large‐scale graphite recycling program, focusing on the purification and reuse of high‐purity graphite products. In the United States, Semco Carbon and Graphite One Inc. are establishing integrated supply chains for reclaimed graphite. Semco provides both graphite manufacturing and reclamation services, while Graphite One is recovering graphite from spent electrodes and machining residues to build a sustainable domestic anode supply chain. In Japan, Toyo Tanso Co., Ltd. has constructed advanced regeneration lines featuring high‐purity processing and closed‐loop recycling.

Globally, the market for recycled graphite remains small but is expanding rapidly. The global graphite recycling market was valued at ≈US $53.9 million in 2023 and is projected to reach US $127.3 million by 2033 (Allied Market Research, 2024). Although specific data on the annual tonnage of recycled graphite are limited, the U.S. Geological Survey notes that “information on the quantity and value of recycled graphite is not available” and industrial pilot plants are scaling up. For instance, Tozero GmbH and American Battery Technology Company (ABTC) have reported recovery efficiencies exceeding 80% and successful remanufacturing of battery cells using fully recycled graphite (Fastmarkets, 2024; ABTC, 2024).

Current industrial strategies mainly follow hydrometallurgical and direct‐recycling pathways. Hydrometallurgical routes involve chemical leaching and heat restoration, while direct recycling preserves graphite microstructure through solvent‐assisted delamination, binder removal, and low‐temperature reactivation. These developments supported by government initiatives such as the U.S. Department of Energy's graphite recycling programs, which represent the transition from laboratory scale research to commercial‐scale implementation in the emerging circular graphite economy.

### 3.2 Direct Recycling Strategies

In the industrial field, several companies have begun implementing direct recycling strategies to recover and reuse graphite anodes while minimizing structural damage. ABTC has established a physical–chemical process to separate and purify graphite for reuse, supported by the U.S. Department of Energy. Ascend Elements has developed its Hydro‐to‐Anode process to obtain high‐purity (≈99.9%) recycled graphite from spent batteries and production residues. Princeton NuEnergy (PNE) has launched a pilot facility that applies mechanical and thermal delamination to recover graphite coatings from copper foils. These industrial efforts demonstrate the feasibility of solvent assisted, energy‐efficient, and closed‐loop direct recycling routes for graphite regeneration.

Recent academic research further supports these industrial developments. Bai et al.^[^ ^37^ ^]^ developed an ethylene‐glycol‐based delamination route that rapidly separates electrode layers within seconds at low temperature without altering the graphite morphology or damaging current collectors, and Ahuis et al.^[^ ^38^ ^]^ extended this concept to industrial electrode scrap, achieving recovery yields of 96% by applying mechanical stress in green solvents and enabling direct reuse of the recovered suspension for new electrode fabrication with negligible capacity loss. Trebeck et al.^[^ ^39^ ^]^ reported an aqueous delamination process in which water, temperature, and ultrasonic energy were tuned to completely remove the active layer from copper foils without chemical reagents, while De Vita et al.^[^ ^40^ ^]^ introduced a froth‐flotation‐assisted process using bio‐derived surfactants and mild organic acids to purify graphite from black mass with more than 96% yield and 99.6% purity. In addition to these separation and purification efforts, He et al.^[^ ^41^ ^]^ developed a calcium‐silicate‐coating strategy that forms a stable amorphous/crystalline interfacial layer on recycled graphite, while Wei et al.^[^ ^42^ ^]^ demonstrated that direct recycling can cut energy consumption by nearly 80% and reduce greenhouse gas emissions by over 90% compared with pyrometallurgical and hydrometallurgical routes. Collectively, these industrial and academic efforts show that direct recycling is evolving from simple material recovery toward high‐value graphite regeneration, offering an energy‐efficient and sustainable route for circular utilization of anode materials.

### 3.3 Hydrometallurgical Recycling Strategies

Hydrometallurgy, or wet metallurgy, is a widely used technique for extracting and recovering valuable metals from ores, industrial waste, or end‐of‐life batteries using aqueous solutions such as acids, alkalis, or salts. This process typically involves leaching, solution purification, and metal recovery through methods like solvent extraction or electrowinning. Compared to traditional pyrometallurgy, hydrometallurgy offers advantages such as lower energy consumption, reduced hazardous emissions, and better control over reaction conditions. In LIB recycling, hydrometallurgical methods are extensively employed to recover valuable metals such as lithium, cobalt, nickel, and manganese from spent electrodes. The acid leaching process usually employs inorganic acids such as H_2_SO_4_,^[^ ^43^, ^44^, ^45^, ^46^, ^47^ ^]^ HNO_3_,^[^ ^45^ ^]^ H_3_BO_3_,^[^ ^48^ ^]^ H_3_PO_4,_^[^ ^49^ ^]^ and HCl,^[^ ^8^ ^,^ ^50^, ^51^ ^]^ while organic acids like citric acid^[^ ^35^ ^]^ offer a greener alternative. Owing to their high selectivity, scalability, and environmental compatibility, hydrometallurgical routes represent a practical and sustainable strategy for closed‐loop LIB recycling.

**Figure 4:**
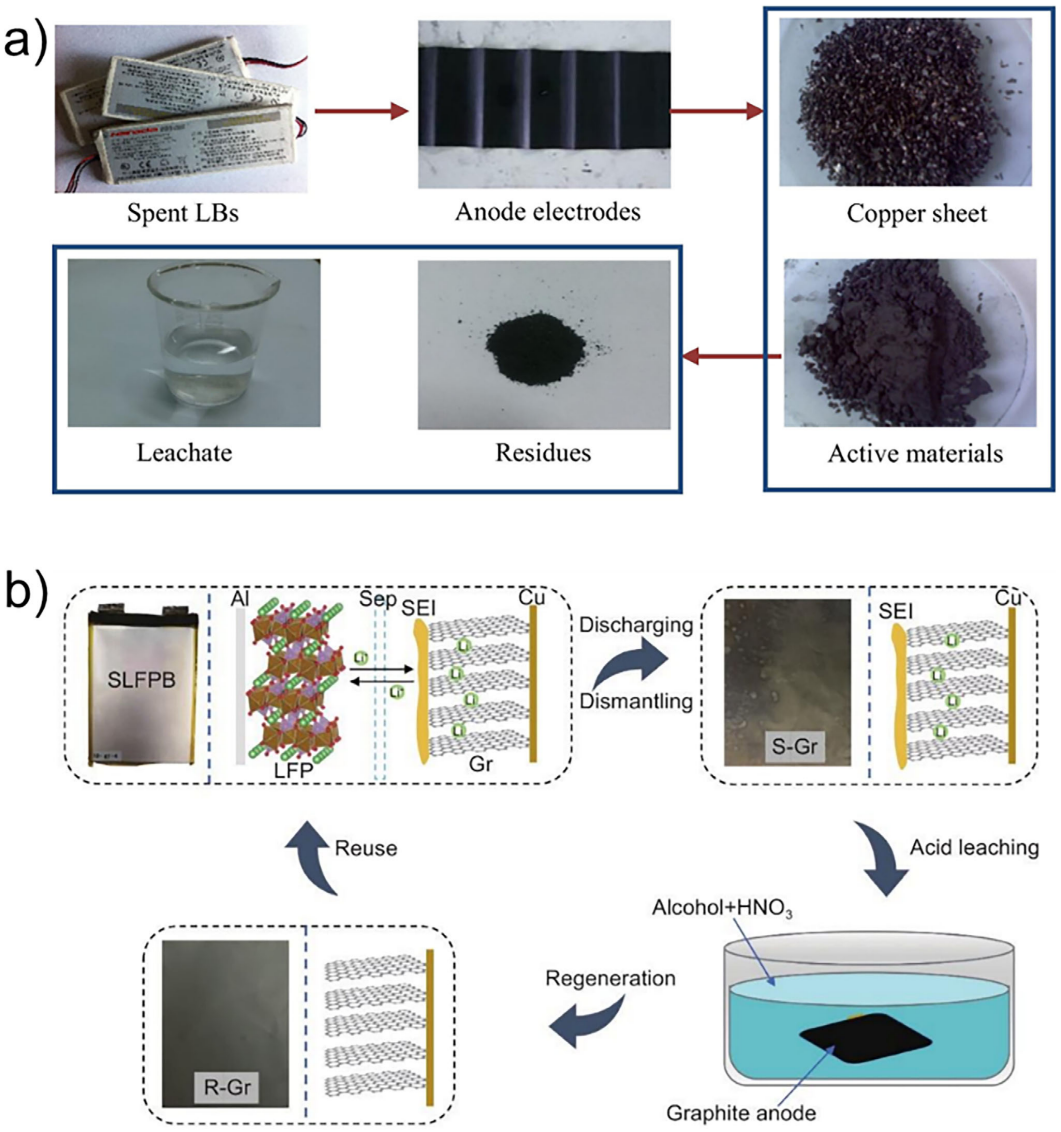
(a) The disassembly and recycling process of anode active material from spent LIBs. Reproduced with permission.^[^ ^50^ ^]^ Copyright 1989, Elsevier. (b) Schematic of the direct regeneration of graphite anode. Reproduced with permission.^[^ ^52^ ^]^ Copyright 2006, Elsevier.

Guo et al.^[^ ^50^ ^]^ demonstrated that HCl extraction efficiently removes lithium, achieving 99.4% recovery at 80 °C, while resulting in a better graphite crystal structure (**Figure** **4**a). Yang et al.^[^ ^51^ ^]^ developed an integrated process combining lithium recovery and graphite regeneration from spent LIBs through two‐stage calcination followed by mild HCl leaching, achieving nearly 100% removal of metallic impurities and high‐purity Li_2_CO_3_ recovery (>99%). Li et al.^[^ ^53^ ^]^ developed a water‐based leaching route, achieving complete graphite exfoliation from copper foil and >92% lithium leaching without hazardous by‐products. Natarajan et al.^[^ ^54^ ^]^ demonstrated an integrated recycling process in which diluted HNO_3_ simultaneously purified graphite and dissolved copper foil, the latter converted into Cu‐based MOFs and CuO. Xu et al.^[^ ^52^ ^]^ proposed a HNO_3_/ethanol washing method to remove the degraded SEI without separating the graphite from the copper, thereby producing smoother graphite surfaces with fewer residuals (Figure 4b). Beyond inorganic acids, citric acid based hydrometallurgical processes have also been shown to provide high efficiency and environmental compatibility. Yang et al.^[^ ^35^ ^]^ achieved a high leaching rate of 97.58% for Li‐ion and transition‐metal removal from spent anodes via citric acid leaching under mild conditions, while maintaining the layered graphite structure.

Hydrometallurgical processes are effective in removing impurities such as residual metals, lithium compounds, and electrolyte residues from spent graphite, thereby enhancing its electrochemical performance. However, leaching alone is insufficient to restore the graphite's structure for direct reuse in LIBs due to residual defects and crystalline degradation. As a result, post‐treatment steps such as high‐temperature calcination are often required.

### 3.4 Hybrid Hydro‐ and Pyrometallurgical Recycling Approaches

While leaching efficiently removes metals, lithium compounds, and electrolyte residues from spent graphite, it fails to repair intrinsic structural defects. Conversely, high‐temperature calcination restores the graphite lattice and removes binders but suffers from high energy consumption and limited metal removal capability. Therefore, combining hydrometallurgical and pyrometallurgical techniques offers a more effective approach to battery graphite recycling.

**Figure 5:**
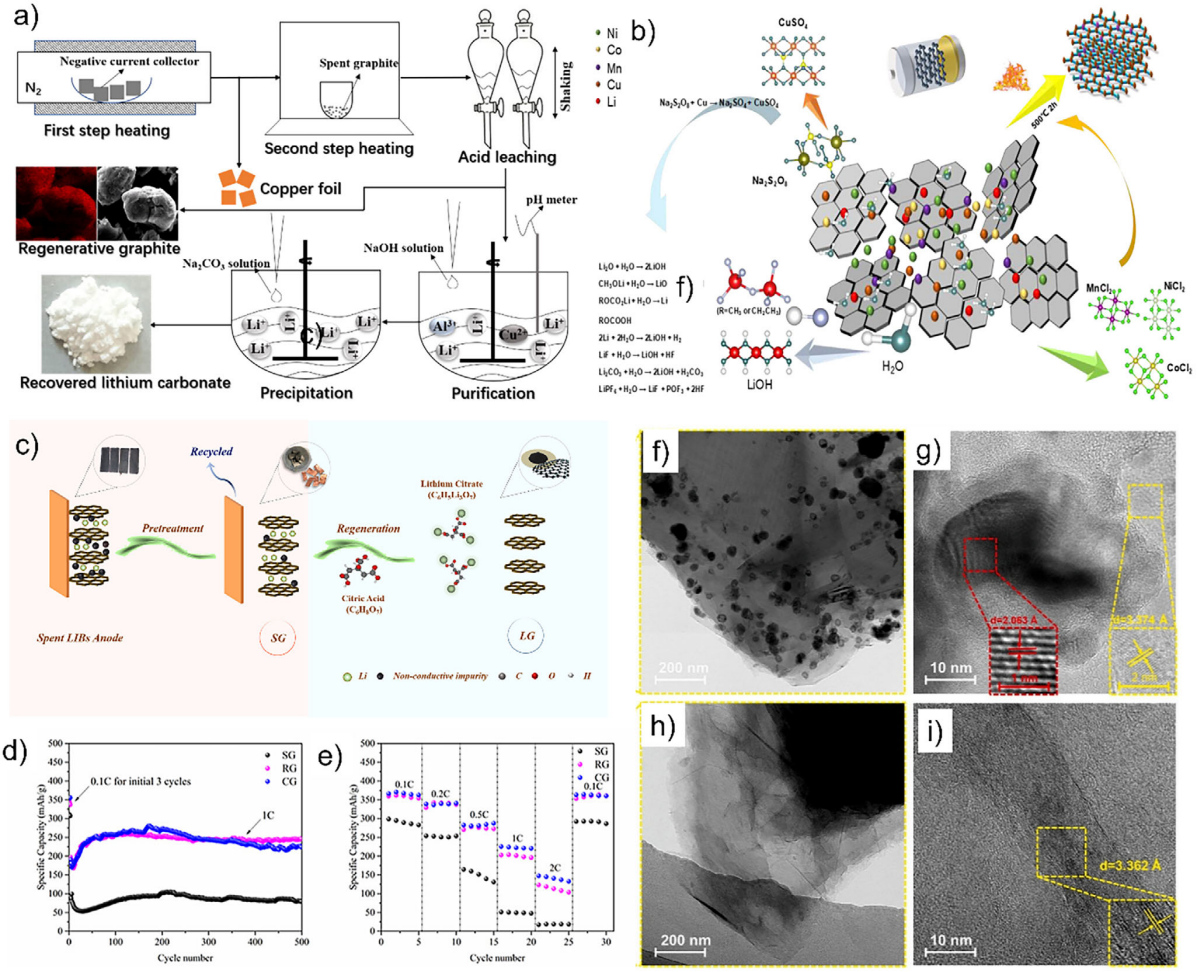
(a) Flow chart of the whole recycling process. Reproduced with permission.^[^ ^51^ ^]^ Copyright 1989, Elsevier. (b) Reaction mechanism diagram in the spent graphite recovery process. Reproduced under the CC BY 4.0 license.^[^ ^55^ ^]^ (c) Schematic diagram of pretreatment and acid leaching process of spent graphite anode materials. Reproduced under the Creative Commons CC BY‐NC‐ND 4.0 license.^[^ ^35^ ^]^ (d) Cycling performances at 1C. (e) Rate performances of SG, RG and CG. Reproduced with permission.^[^ ^56^ ^]^ Copyright 1963, Elsevier. High‐resolution TEM image of SG (f,g), and RG (h,i). Reproduced with permission.^[^ ^57^ ^]^ Copyright 1963, Elsevier.

Yang et al.^[^ ^51^ ^]^ further optimized the process via two‐stage calcination followed by 1.5 M HCl leaching (**Figure** **5**a), removing metallic contaminants and regenerating graphite with high capacity retention (97.9% after 100 cycles). Acid pretreatment effectively removes impurities, with HCl converting residual metals into soluble chlorides (e.g., CoCl_2_, MnCl_2_, NiCl_2_) (Figure 5b).^[^ ^55^ ^]^ Subsequent high‐temperature calcination eliminates organic residues and repairs the graphite lattice, increasing interlayer spacing and restoring electrochemical performance. Another commonly used leaching agent is H_2_SO_4_,^[^ ^7^, ^43^, ^58^, ^59^ ^]^ which effectively removes metal impurities from spent graphite. When combined with subsequent high‐temperature calcination, it facilitates the restoration of the graphite structure. Sometimes, mixed acids (e.g., H_2_SO_4_/HNO_3_) are also employed to enhance the leaching efficiency.^[^ ^45^ ^]^ Some studies also utilize a combined alkali‐acid‐thermal treatment approach to enhance the overall process.^[^ ^60^ ^]^

Yang et al.^[^ ^35^ ^]^ developed an efficient recycling method for spent LIBs graphite using thermal treatment combined with green organic citric acid leaching (Figure 5c). This process effectively removed impurities and recovered lithium, while significantly enhancing the specific capacity, cycling stability, and rate performance of the regenerated graphite (RG). Chen et al.^[^ ^56^ ^]^ proposed an acid leaching–assisted catalytic graphitization method. The spent graphite (SG) was thermally treated and leached with H_2_SO_4_, followed by cobalt nitrate–assisted graphitization at 900 °C under N_2_. After removing Co, the RG delivered 358 mAh g^−1^ at 0.1 C and retained 245.4 mAh g^−1^ after 500 cycles at 1 C (Figure 5d,e), demonstrating effective structural restoration.

Leaching agents are typically organic or inorganic acids, which suffer from high cost and corrosiveness. Zeng et al.^[^ ^57^ ^]^ developed a roasting‐assisted leaching process using waste polyvinyl chloride (PVC), followed by calcination at 1000 °C for 3 h, to successfully synthesize RG. High‐resolution TEM revealed that thermal treatment restored the interlayer spacing of RG to 0.3362 nm, consistent with ideal graphite, while effectively removing copper impurities present in the SG (Figure 5d–g). Recently, Natarajan et al.^[^ ^61^ ^]^ developed an environmentally friendly regeneration strategy for spent LIB graphite using sequential treatments with acid (CH_3_COOH), alkali (KOH), and annealing under N_2_ gas, achieving delithiation capacities of 328–338 mAh g^−1^ with ∼99.9% Coulombic efficiency, comparable to commercial graphite (CG).

### 3.5 Microwave‐Assisted Treatment

Microwave‐assisted thermal strategies offer a promising alternative to conventional heating methods for regenerating spent graphite. Unlike traditional conduction‐based heating, microwave irradiation enables rapid and localized temperature rise by directly interacting with dielectric or conductive materials.^[^ ^62^ ^]^ This selective volumetric heating reduces energy consumption and processing time while minimizing structural damage. In graphite recycling, microwave treatment effectively removes surface impurities, enhances graphitization, and reconstructs internal channels that facilitate lithium‐ion transport, ultimately improving electrochemical performance. These features highlight the potential of microwave technology in efficient and sustainable graphite regeneration. Fang et al.^[^ ^63^ ^]^ introduced a microwave‐assisted calcination process following sulfuric acid curing and leaching, enabling rapid structural restoration and achieving a capacity of 354.1 mAh g^−1^ with 98.3% retention after 60 cycles. Hou et al.^[^ ^64^ ^]^ showed that microwave irradiation effectively enlarged interlayer spacing and created open diffusion sites, enhancing lithium storage and kinetics. This approach enabled a specific capacity exceeding 400 mAh g^−1^, together with improved charge transfer and rate performance. The regeneration process and their correponding electrochemical performances disscussed above are summarized in **Table** **3**.

**Table 3:** Comparison of regeneration processes and electrochemical properties of reused graphite.

Separation process	Cleaning process	Calcination temp/time	Cycling performance (Capacity retention/cycle number/current density)	Refs.
Two‐stage calcination followed by 1.5 M HCl leaching (60 min, S/L = 100 g·L^−1^)	pH adjustment to 7–9 for selective Al/Cu removal, Li recovered as Li_2_CO_3_	‐	97.9%/100/372 mA g^−1^	[51]
Direct regeneration without separating graphite from Cu foil	Acid washing followed by ethanol rinse and drying at 60 °C	‐	289 mAh g^−1^/60 /0.05 A g^−1^	[52]
Stepwise purification using hydrothermal leaching and acid leaching to selectively remove metal impurities (Cu, Li, Co, Mn, Ni) from spent graphite	Hydrothermal Cu removal with Na_2_S_2_O_8_, followed by water leaching of Li and HCl leaching of Co/Mn/Ni; subsequent washing and filtration	500 °C/2 h	372.65 mAh g^−1^/100 /0.5C	[55]
Anodes were stripped, solvent‐cleaned with ethanol, NMP, and DMC, and thermally pre‐treated at 450 °C to remove binders and electrolytes	Hydrometallurgical acid purification using H_2_SO_4_: HNO_3_ = 4: 1 at 95 °C for 4 h to dissolve Cu, F, and electrolyte residues	900 °C/3h	291.7 mAh g^−1^ /140/0.5C	[45]
Spent graphite was detached from Cu foil by alcohol‐assisted ultrasonic stirring, followed by washing with NMP, alcohol, and deionized water to remove binders and electrolyte residues	Acid leaching with concentrated H_2_SO_4_ at 80 °C for 5 h	800 °C/8h	427.9 mAh g^−1^ /200/0.5C	[7]
Water leaching to separate graphite from dissolved Li/Co/Ni/Mn/Al/Cu species	Aqueous H_2_SO_4_ leaching and rinse with deionized water	1500 °C/2h	99.7%/50/0.1C	[58]
Sieving (<500 µm) + acid leaching (2 M H_2_SO_4_ + 2 vol% H_2_O_2_, 60 °C 3 h)	Acidified water rinse	800 °C/1h	80%/100/1C	[59]
Manual disassembly, stripping anode graphite, acid leaching in 200 g L^−1^ H_2_SO_4_ (95 °C, 4 h)	Washed with deionized water until neutral	700‐1500 °C/2h	345.5 mAh g^−1^ /100/0.1C	[43]
Manual disassembly, discharge in NaCl solution, calcination at 450 °C for 1.5 h (binder/electrolyte removal), 500 °C 1 h (oxidation of residual Cu/Al)	Citric acid leaching (0.2 m, 90 °C, 50 min, S/L = 1:50 g mL^−1^, 300 rpm), filtration and drying	450‐500 °C/1‐1.5h	330 mAh g^−1^/80/0.5C	[35]
Mechanical crushing, sieving (<500 µm), hydrometallurgical leaching (2 M H_2_SO_4_ + 2 g L^−1^ Fe^3+^, 60 °C, 3 h)	Residue washed with acidified water (pH 2), dried for pyrolysis	800 °C/1h	97.7%/70/0.2 C	[60]
Sulfuric acid curing (200 °C for 2 h), acid leaching with 200 g·L^−1^ H_2_SO_4_ (90 °C, 4 h)	Filtration and washing with deionized water to pH 7, drying at 80 °C for 8 h	Microwave calcination at 800 °C for 1 h under Ar (200 °C·min^−1^ heating rate)	≈350 mAh g^−1^/60/0.1C	[63]
Manual separation of graphite from spent LIBs, preliminary heating at 550 °C for 60 min in air	Acid leaching with 1 M H_2_SO_4_ (150 mL), washed repeatedly with deionized water, dried at 80 °C overnight	Microwave irradiation in air at 800 W for 15–40 s	372.92 mAh g^−1^ /100 /0.1 A g^−1^	[64]
Sorting; discharge in 10% NaCl for 24 h; drying; shredding; pulverization; sieving through 100 µm; bioleaching using acidithiobacillus ferrooxidans at 100 g L^−1^, 30 °C and 160 rpm; filtration every 2 h for three cycles to obtain the bioleaching residue	Cleaning with 1 M H_2_SO_4_ or 1 M citric acid at 90 °C for 5 h, followed by 16 h standing at room temperature; washing with deionized water three times; drying at 60 °C for 24 h	750 °C/5h	412 mAh g^−1^/200/0.1 A g^−1^	[65]
Manual removal from Cu foil; DMC rinsing; PVDF removal in NMP (80 °C, 5 h); centrifugation; DI‐water washing; vacuum‐drying at 80 °C, 12 h	NMP cleaning + DI water washing; optional 5 wt% H_3_BO_3_ coating (80 °C, 12 h) for B‐doping	750–1050 °C/1h	333 mAh g^−1^/100/C/3	[48]

### 3.6 New Approaches: Ultra‐Fast Thermal Shock/Treatment

Recycling spent graphite from spent LIBs is typically a time‐consuming and energy‐intensive process due to complex impurity removal and structural degradation. Traditional thermal treatments require prolonged heating at high temperatures, limiting scalability. Ultra‐rapid heat treatment such as flash Joule heating (FJH) technology, as an emerging technique based on rapid resistive heating, offers an efficient and scalable alternative. This approach effectively eliminates residual binders and inorganic contaminants while recovering the graphitic crystallinity, thereby offering a scalable and sustainable strategy for high‐quality graphite regeneration.

**Figure 6:**
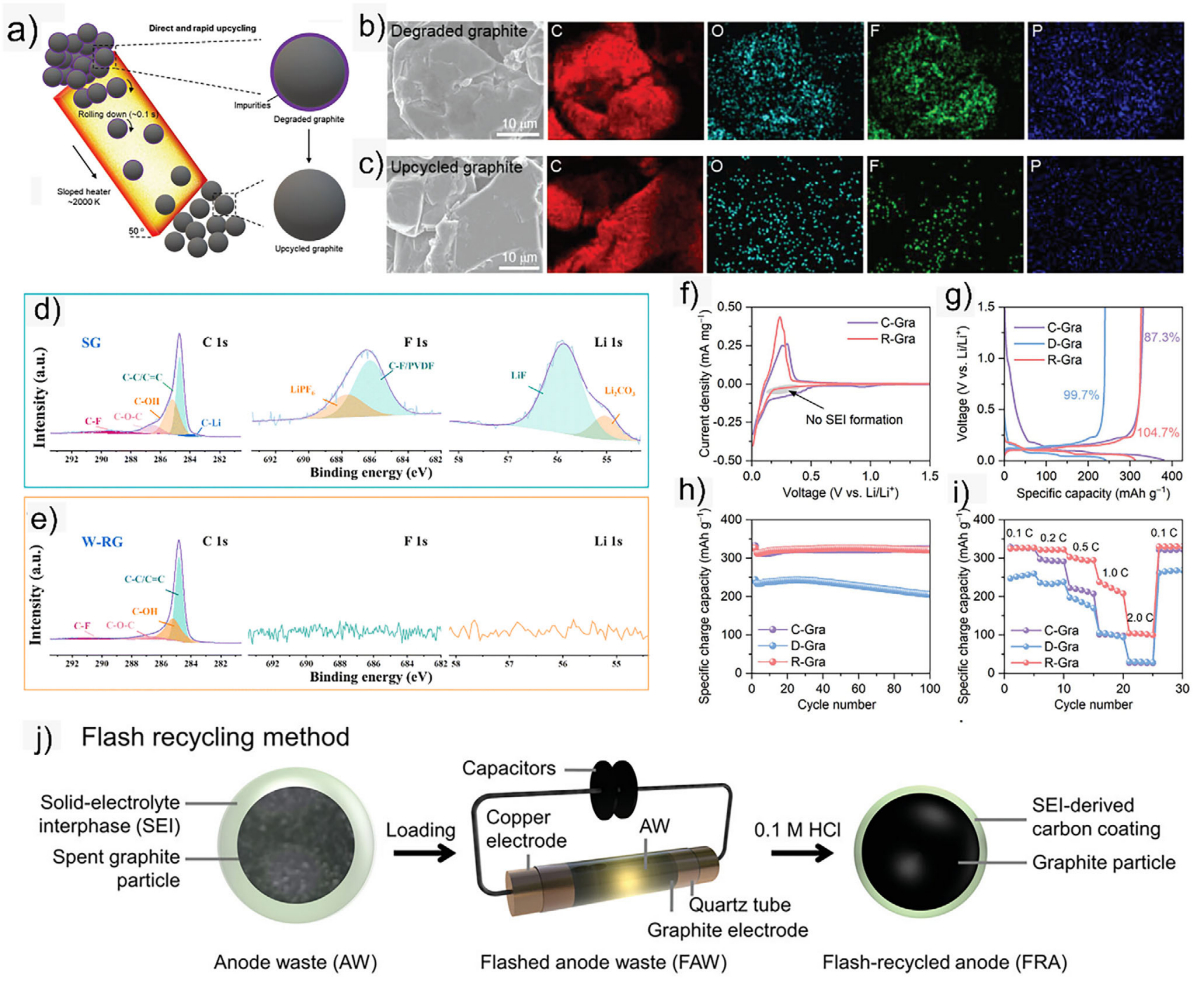
(a) Direct and rapid high‐temperature upcycling of graphite anodes. SEM images and elemental mappings of the (b) degraded and (c) upcycled graphite. Reproduced under the CC BY 4.0 license.^[^ ^68^ ^]^ Survey XPS and high‐resolution C 1s, F 1s, and Li 1s spectra for (d) RG and (e) W‐RG. Reproduced with permission.^[^ ^73^ ^]^ Copyright 2016, Royal Society of Chemistry. (f) The initial cyclic voltammetry (CV) curves at a scan rate of 0.1 mV s^−1^. (g) The initial charge–discharge profiles at 0.1 C. (h) Cycling performance at 0.2 C (0.1 C for the initial two cycles). (i) Rate performance at 0.1 to 2 C. (j) Schematic of flash recycling of anode waste. Reproduced with permission.^[^ ^70^ ^]^ Copyright 2023, Wiley‐VCH.

Zheng et al.^[^ ^66^ ^]^ proposed a rapid thermal shock (RTS) process, achieving fast impurity removal and lattice restoration. Xiong et al.^[^ ^67^ ^]^ further developed FJH, an ultra‐fast process (>3000 K) that restores graphite within milliseconds, which effectively removes impurities and improves graphitization. By employing a sloped carbon heating device, a continuous high‐temperature heating (≈2000 K) process can be achieved in an extremely short time (≈0.1 s, **Figure** **6**a).^[^ ^68^ ^]^ This approach effectively removed surface impurities and optimized graphitization, as confirmed by SEM–EDS analysis (Figure 6b,c), where upcycled graphite (UG, referring to the conversion of spent graphite into higher‐value anode materials) exhibited a smooth, impurity‐free surface. Ji et al.^[^ ^69^ ^]^ introduced a millisecond‐scale rapid heating strategy (≈1900 K, ≈150 ms) that transforms thick organic–inorganic SEI layers into compact inorganic‐rich films (≈10–30 nm), as illustrated in Figure 6f. This process retains active Li, leading to an exceptionally high initial Coulombic Efficiency (ICE) of 104.7% in half‐cell tests and improving both the initial CE (98.8% vs 83.2%, Figure 6g) and energy density (309.4 vs 281.4 Wh kg^−1^) of full cells. The robust inorganic SEI, comprising LiF, Li_2_CO_3_, and Li_2_O, enhances mechanical durability and ionic conductivity (Figure 6h,i).

Chen et al.^[^ ^70^ ^]^ further advanced FJH (≈2850 K in <1 s, Figure 6j), efficiently decomposing the SEI and protective carbon shell. They also employed 0.1 M HCl to leach and recover valuable metals such as Li, Co, Ni, and Mn. The high‐temperature shock (HTS) could create defect‐rich graphite (DRG) through ultrafast heating and cooling.^[^ ^71^ ^]^ The introduced defects reduced strain energy and enhanced Li^+^ diffusion, enabling a high‐rate capacity of 323 mAh g^−1^ at 2 C. The economic and environmental benefits of such techniques are also notable. Shang et al.^[^ ^72^ ^]^ reported that FJH reduced regeneration costs to $7.75/kg and significantly lowered greenhouse gas emissions, supporting a circular economy model for LIB recycling. And the recycling process and performances based on ultra‐fast thermal shock/treatment are summarized in **Table** **4**.

Overall, each recycling methodology exhibits distinct advantages and limitations. Pyrometallurgical routes are technically mature and suitable for large‐scale processing, but their high energy consumption and irreversible degradation of graphite limit reuse potential. Hydrometallurgical methods enable high‐purity recovery through chemical leaching and purification, yet they involve complex liquid handling and environmental

**Table 4:** Comparison of electrochemical properties of reused graphite with ultra‐fast thermal shock/treatment.

Recycling process	Cycling performance (Capacity retention/cycle number)	Refs.
Degraded graphite directly regenerated under ultrafast heating (≈3000 °C, <1 s) in inert atmosphere, achieving impurity removal and lattice restoration.	320 mAh g^−1^/500	[68]
Flash recycling of graphite anodes: spent graphite rapidly regenerated by flash Joule heating (≈3000 K, <1 s) without chemical reagents, achieving impurity removal and graphitization.	335.9 mAh g^−1^/100	[69]
Degraded graphite directly regenerated under ultrafast heating (≈3000 °C, <1 s) in inert atmosphere, achieving impurity removal and lattice restoration.	320 mAh g^−1^/500	[68]
Spent graphite thermally regenerated and modified by SEI reconstruction to encapsulate active Li and improve interfacial stability.	321.5 mAh g^−1^ /100	[69]
Spent graphite regenerated at 1000 °C for 5 s under Ar by applying 40 V to carbon paper; organic residues decomposed and amorphous carbon layer formed.	80 mAh g^−1^/600	[66]
Spent graphite subjected to ultrafast heating and cooling (≈2000–2500 °C, few seconds) to form defect‐rich recycled graphite with recovered layered structure.	282 mAh g^−1^/1000	[71]

Burdens associated with acid consumption and wastewater treatment. In contrast, direct recycling strategies retain the graphite microstructure and reduce both energy demand and carbon footprint, though their scalability and cost‐effectiveness still require optimization. Hybrid and microwave‐assisted processes offer a promising balance between efficiency and sustainability by combining moderate thermal input with selective chemical activation. Meanwhile, ultra‐fast thermal shock treatments such as flash Joule heating represent an emerging route that achieves rapid binder removal and defect healing within seconds, offering high efficiency and scalability but still facing challenges in temperature uniformity and surface control. Together, these methodologies provide complementary pathways for sustainable graphite regeneration, and their comparative understanding is essential for selecting appropriate recycling routes based on performance, environmental, and industrial considerations.

### 3.7 Spent Graphite for Different Applications

Beyond its reuse as an anode material for LIBs, recycled graphite has been increasingly explored for applications in catalysis,^[^ ^74^, ^75^ ^]^ reduced graphene oxide (rGO), polymer composites,^[^ ^76^, ^77^ ^]^ and supercapacitor.^[^ ^78^ ^]^ Recycled graphite can serve as an efficient catalyst carrier owing to its high conductivity and chemical stability. Zhao et al.^[^ ^79^ ^]^ demonstrated that regenerated graphite‐supported peroxymonosulfate (PMS) systems effectively degraded organic pollutants within minutes under visible light, providing a sustainable pathway for wastewater treatment. Wang et al.^[^ ^80^ ^]^ prepared reactive rGO from waste graphite, which exhibited excellent catalytic ozonation activity for the degradation of organic pollutants. The study revealed that a higher density of structural defects significantly enhanced catalytic performance by facilitating the decomposition of ozone molecules into reactive oxygen species (ROS) at vacancy and edge sites of the graphene framework. These findings emphasize the key role of defect engineering in optimizing the catalytic behavior of recycled graphene‐based materials. In carbon material synthesis, spent graphite can be transformed into rGO and carbon hollow spheres (CHS) through environmentally benign oxidation–reduction or exfoliation processes. Zhang et al.^[^ ^81^ ^]^ reported a green route for preparing rGO from spent graphite via a modified Hummers method, which delivered excellent capacitive behavior in supercapacitors. Natarajan et al.^[^ ^82^ ^]^ developed a template‐free synthesis of CHS and rGO hybrids from spent graphite, achieving high CO_2_, N_2_ and H_2_ adsorption capacities and demonstrating their potential in gas storage. Divya et al.^[^ ^83^ ^]^ further fabricated high‐energy dual‐carbon lithium‐ion capacitors using recovered graphite, achieving an energy density of 185.5 Wh kg^−1^ and stable cycling over 2000 cycles, demonstrating the potential of recycled graphite for cost‐effective and durable energy storage. Yang et al.^[^ ^84^ ^]^ first introduced a redox‐based approach to recover graphite anode materials by converting spent graphite into graphene. A modified Hummers method was used to oxidize graphite, followed by reduction with vitamin C to obtain graphene with a well‐defined layered structure. Most oxygen‐containing functional groups were effectively removed during reduction, restoring the structural integrity of the carbon framework. Recycled graphite has also been used to produce polymer‐graphite nanocomposite thin films. Natarajan et al.^[^ ^85^ ^]^ synthesized polypropylene (PP)/polyethylene (PE) and graphite composite thin film (PP/GRx and PE/GRx) from recovered graphite and polymer residues, where the tensile strength increased from 3.0–3.4 to over 33 MPa and the electrical conductivity improved by 5–6 orders of magnitude. Such composites exhibit high crystallinity, mechanical robustness, and flexibility, suitable for conductive films and flexible electronic applications. Together, these studies demonstrate that regenerated graphite can be effectively upcycled into high‐value materials, including rGO, carbon nanostructures, polymer composites, and dual‐carbon electrodes, expanding its utilization from conventional battery reuse to multifunctional energy and environmental applications.

## 4 Functional Modifications of Regenerated Graphite

### 4.1 Surface Engineering

Through various recycling methods, the structure and properties of spent graphite can be recovered. However, the recycled graphite still faces volume expansion and severe decomposition of the electrolyte on its surface during lithiation.^[^ ^88^ ^]^ Applying a protective layer or artificial SEI to the surface of graphite can effectively stabilize its structure and even enhance its fast‐charging performance.^[^ ^89^, ^90^ ^]^

**Figure 7:**
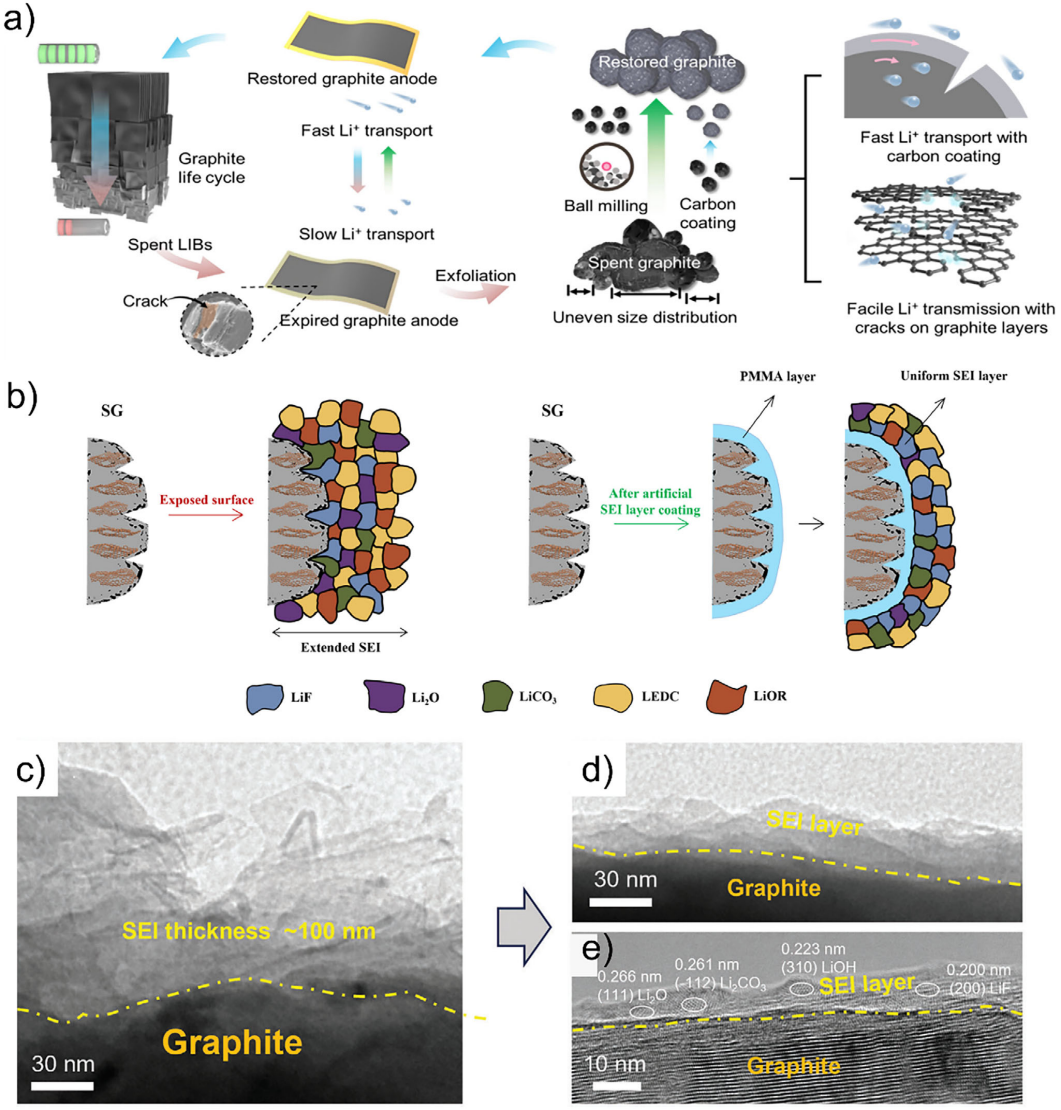
(a) Schematic of the regeneration and carbon coating process. Reproduced with permission.^[^ ^86^ ^]^ Copyright 2024, American Chemical Society. (b) Schematic diagram of natural and artificial SEI formation on the surface of recycled graphite. Reproduced with permission.^[^ ^87^ ^]^ Copyright 2023, Copyright 1998, Elsevier. TEM images of (c) degraded graphite (D‐Gra) and (d) regenerated graphite (R‐Gra). (e) The high‐resolution TEM (HRTEM) images of R‐Gra. Reproduced with permission.^[^ ^69^ ^]^ Copyright 1998, Wiley‐VCH.

Carbon‐based coatings are the most widely used coating strategy. Hou et al.^[^ ^91^ ^]^ combined pre‐oxidation, acid leaching, and pitch/resin‐derived carbon coating to form a porous core–shell structure that improved conductivity and Li‐ion kinetics. The coated graphite achieved 441 mAh g^−1^ at 50 mA g^−1^ and 95% retention over 200 cycles. High‐temperature re‐graphitization followed by carbon coating further enhanced ICE and rate capability.^[^ ^92^ ^]^ Luo et al.^[^ ^93^ ^]^ employed a Mg‐based catalyst and saccharose to generate a dense turbostratic carbon coating on the surface of graphite, which effectively repaired structural cracks, suppressed interfacial side reactions, and enhanced the kinetics of Li^+^ transport. Chen et al.^[^ ^86^ ^]^ proposed a strategy for regenerating and upgrading spent graphite via interface regulation using styrene–maleic anhydride copolymer (SMA) (**Figure** **7**a). They employed a simple ball‐milling homogenization and low‐temperature sintering carbonization process to construct a uniform carbon coating derived from SMA on the surface of spent graphite. This carbon coating was critical for creating active Li⁺ sites on the graphite basal plane and enhancing surface isotropy, thus facilitating rapid Li⁺ migration and improving electrochemical performance. Carbon coatings derived from various precursors, including phenolic resin.^[^ ^47^ ^]^ PVDF,^[^ ^94^ ^]^ asphalt,^[^ ^95^ ^]^ glucose,^[^ ^96^ ^]^ and pitch,^[^ ^97^ ^]^ have also been demonstrated to significantly improve the electrochemical performance of graphite.

The SEI plays a critical role in the performance of graphite anodes, and SEI engineering has recently attracted considerable research attention.^[^ ^98^, ^99^ ^]^ Artificial SEI layers can protect graphite from electrolyte‐induced degradation and help stabilize the electrode structure, thereby enhancing its cycling stability. By deliberately tailoring the SEI composition, Li^+^ desolvation can be facilitated and Li^+^ transport within the SEI accelerated, enabling fast charge/discharge capabilities. Da et al.^[^ ^87^ ^]^ demonstrated the application of a poly(methyl methacrylate) (PMMA) artificial SEI coating on spent graphite (SG) (Figure 7b). The PMMA layer effectively protected damaged graphite surfaces, enhanced surface compatibility, and improved electrochemical performance. This artificial PMMA SEI reduced electrolyte decomposition, promoting uniform formation of a thin, stable natural SEI. Ji et al^[^ ^69^ ^]^ developed a fast‐heating strategy (∼1900 K for ≈150 ms) to effectively regenerate degraded graphite (D‐Gra) by reconstructing its surface SEI layer. This rapid thermal treatment converted the originally thick and loose SEI (≈100 nm, Figure 7c) into a thin and compact layer (≈10–30 nm), enriched with inorganic components such as LiF, Li_2_CO_3_, and Li_2_O. HRTEM analysis confirmed the presence of these stable inorganic species, which contributed to improved air stability by preserving the embedded lithium (Figure 7d,e). As a result, the regenerated graphite exhibited enhanced Coulombic efficiency, stable cycling, and higher energy density in full‐cell configurations. Various artificial SEI layers such as polydopamine (PDA),^[^ ^100^, ^101^ ^]^ metal oxides,^[^ ^102^, ^103^ ^]^ Li_3_P^[^ ^89^ ^]^ and polymer^[^ ^104^, ^105^ ^]^ have also demonstrated their effectiveness in stabilizing the electrode–electrolyte interface.

### 4.2 Tailoring Materials Properties via Structure Design and Doping

The graphite structure plays a crucial role in determining its performance. By tuning the porous architecture, adjusting interlayer spacing, engineering defects, and doping with heteroatoms, the Li^+^ transport properties can be significantly enhanced. Porous graphite can be successfully synthesized by employing pore‐forming agents such as MoO_x_,^[^ ^107^ ^]^ Ni,^[^ ^108^ ^]^ and KOH.^[^ ^109^ ^]^ These pores and interconnected channels significantly enhance active sites and facilitate rapid Li^+^ transport. The graphite interlayer spacing of ≈3.35 Å leads to sluggish Li^+^ intercalation and deintercalation. And expanding the interlayer spacing can effectively enhance the rate‐performance of graphite.

**Figure 8:**
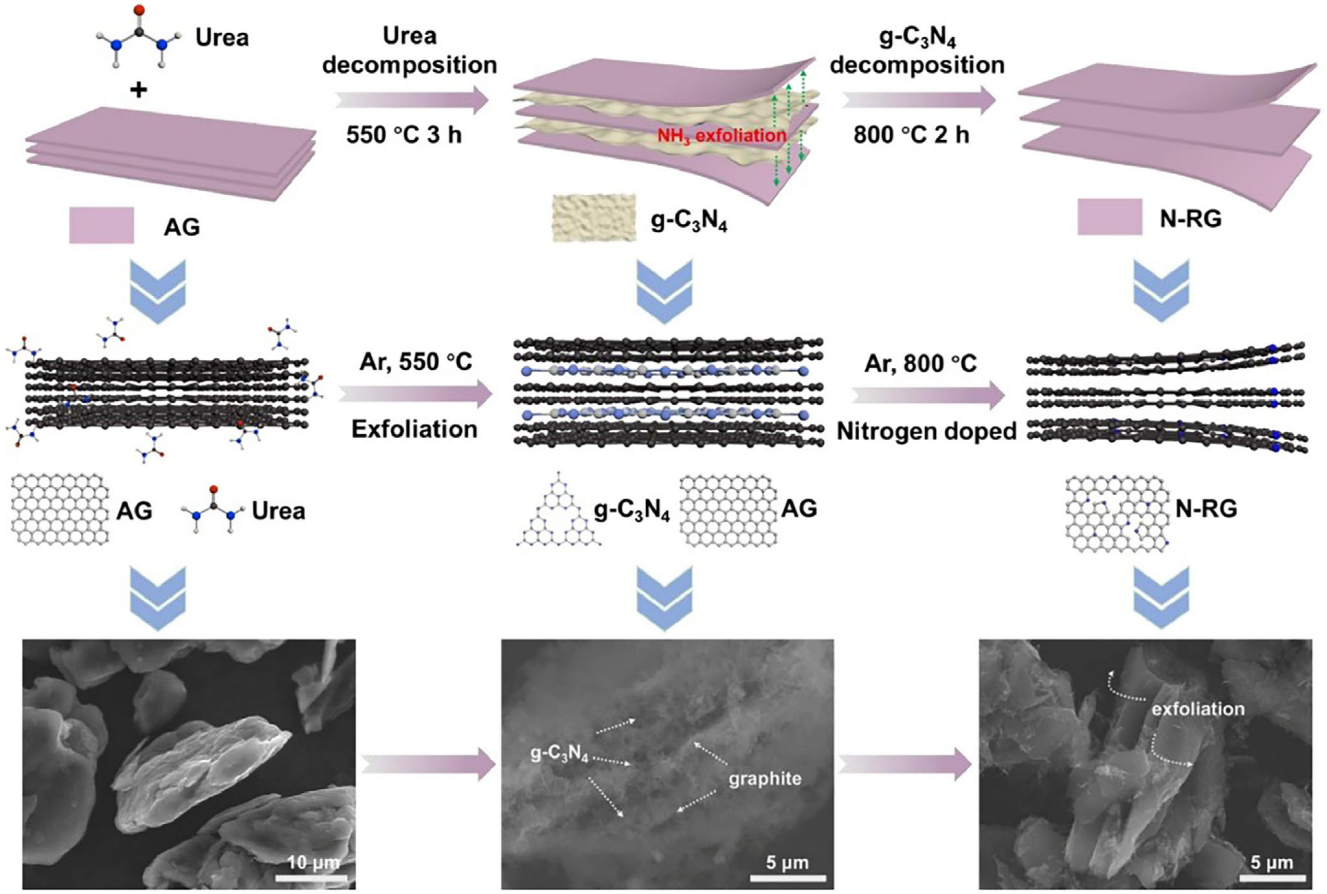
Schematic illustration of the synthesis of novel regenerated graphite (N‐RG) with nitrogen doping and enlarged interlayer spacing. Reproduced with permission.^[^ ^106^ ^]^ Copyright 2022, Elsevier.

Adjusting the interlayer spacing of graphite and doping strategies have been explored to tailor the graphite structure. Yang et al.^[^ ^110^ ^]^ developed a phosphoric acid leaching–calcination method that introduced phosphorus into the graphite lattice, forming C─O─P and C─P bonds and expanding the interlayer spacing from 0.335 to 0.361 nm. Xu et al.^[^ ^106^ ^]^ applied a one‐step gas‐phase nitrogen doping and exfoliation strategy, increasing interlayer spacing (0.337 to 0.348 nm) and enabling fast‐charging performance (143.5 mAh g^−1^ at 4 A g^−1^) (**Figure** **8**). Markey et al.^[^ ^48^ ^]^ proposed boron doping with thermal annealing, healing defects and stabilizing graphite structure, effectively narrowing the performance gap with pristine graphite. These modifications enhanced wettability and Li^+^ transport, resulting in superior capacity and cycling stability.

### 4.3 Development of Composite Materials with Hybrid Components

**Figure 9:**
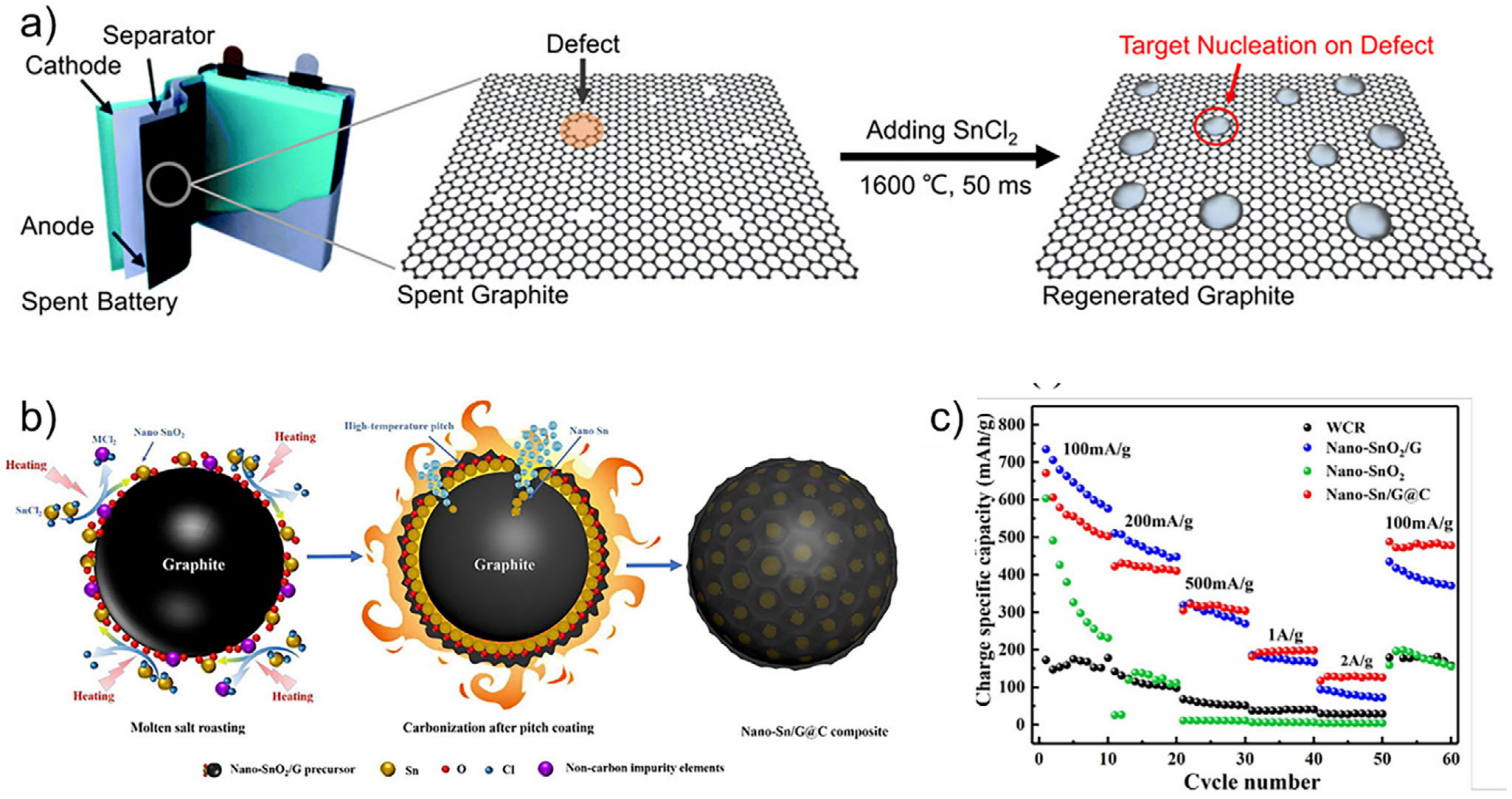
(a) Schematic of targeted regeneration and upcycling of spent graphite via flash heating. Reproduced under the CC BY 4.0 license.^[^ ^88^ ^]^ (b) Schematic of the synthesis of nano‐SnO_2_/G precursor and nano‐Sn/G@C composite anode. (c) Rate performance of waste carbon residue (WCR), nano‐SnO_2_/G, nano‐Sn/G@C, and nano‐SnO_2_. Reproduced with permission.^[^ ^111^ ^]^ Copyright 2023, Elsevier.

Graphite exhibits a relatively low theoretical capacity of only 372 mAh g^−1^, which is insufficient to meet the demands for high‐energy‐density applications. Therefore, considerable efforts have been devoted to combining graphite with high‐capacity alternatives, including Si,^[^ ^112^, ^113^ ^]^ P,^[^ ^114^ ^]^ black phosphorus (BP),^[^ ^115^ ^]^ Ge^[^ ^116^ ^]^ and Sn,^[^ ^117^ ^]^ to enhance electrochemical performance. Ruan et al.^[^ ^118^ ^]^ fabricated Si–graphite composites using porous regenerated graphite, achieving 434.1 mAh g^−1^ at 500 mA g^−1^ and 92.5% retention over 300 cycles. Cheng et al.^[^ ^88^ ^]^ employed FJH (1600 °C, 50 ms) to thermally reduce the preloaded SnCl_2_ into molten Sn, which preferentially nucleated at defect sites on the graphite (**Figure** **9**a). This defect‐targeted nucleation and regeneration strategy enabled the instantaneous healing of spent graphite. Similarly, a molten‐salt approach has been proposed for regenerating waste graphite into high‐capacity nano‐Sn/graphite@C (nano‐Sn/G@C) composite anodes (Figure 9b).^[^ ^111^ ^]^ This method not only yields smaller Sn nanoparticles compared to conventional hydrothermal synthesis but also offers greater industrial scalability. By utilizing the porous and oxidized structure of WCR, nano‐SnO_2_/G precursors are formed and subsequently reduced to nano‐Sn, which intercalates into the graphite matrix, enhancing structural buffering and electrochemical performance (Figure 9c).

**Figure 10:**
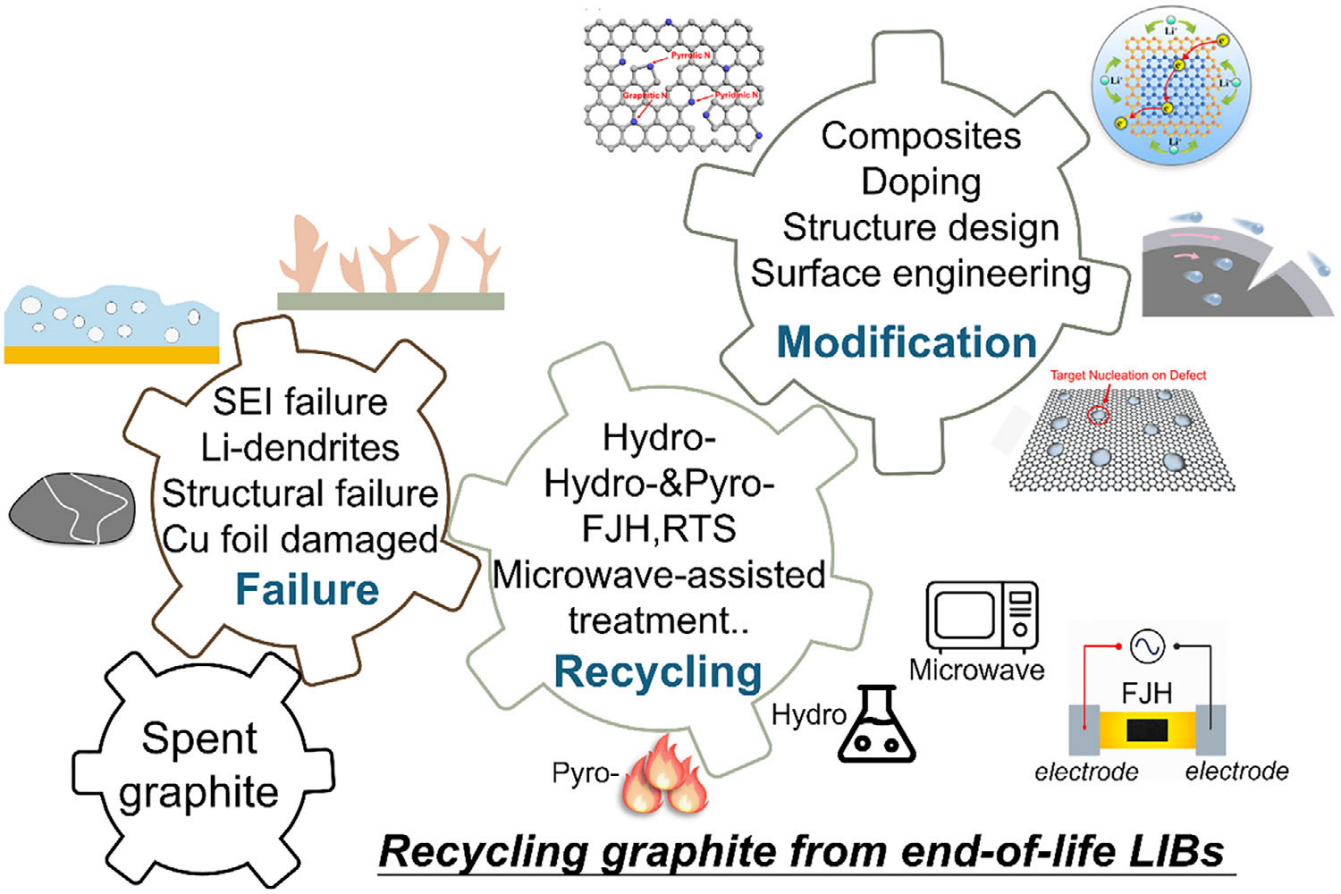
The failure mechanisms, recycling and functional modification of spent graphite anode.

## 5 Conclusion and Outlook

Recycling and regenerating graphite from spent LIBs is vital for establishing a sustainable and circular battery industry. The growing demand for graphite and the environmental burden of spent batteries have driven intensive efforts to recover and upcycle anode materials. However, several challenges persist, including structural degradation during cycling, SEI failure, and surface contamination by binders, conductive additives, and metallic residues, which complicate purification and regeneration (**Figure** **10**). Conventional high‐temperature and acid‐leaching routes are energy‐intensive and generate secondary waste, while direct recycling offers a low‐energy and structure‐preserving alternative that can reduce energy use by nearly 80% and greenhouse gas emissions by over 90%, demonstrating clear sustainability advantages. From a life cycle assessment (LCA), pyro‐ and hydro‐recycling routes generally involve higher energy consumption and greater environmental burdens due to high‐temperature calcination and acid leaching, leading to increased greenhouse gas emissions and water usage. In contrast, direct recycling minimizes chemical input and energy demand by preserving the graphite structure, thereby reducing overall energy consumption and emissions while mitigating secondary pollution. This indicates a clear sustainability advantage of structure‐preserving direct regeneration in the circular graphite economy.

Despite these advances, regenerated graphite often exhibits lower ICE, rate capability, and cycling stability than pristine materials because of lattice distortion and increased interlayer spacing. To address these issues, surface and interface engineering such as carbon coating, artificial SEI construction, and heteroatom doping (P, N, B, or S) can repair structural defects, stabilize electrode–electrolyte interfaces, and enhance ion transport kinetics. Combining regenerated graphite with high‐capacity materials such as silicon or tin may further improve energy density while maintaining durability.

From a practical perspective, reuse of regenerated graphite in LIBs represents the most feasible near‐term route due to process compatibility, low cost, and mature industrial infrastructure. In the longer term, high‐value applications such as sodium‐ion batteries, supercapacitors, catalytic supports, and structural composites provide additional opportunities for upcycling. Future work should focus on developing green, scalable, and cost‐efficient recycling technologies, including organic acid leaching, salt‐assisted purification, and low‐temperature catalytic graphitization, along with standardized industrial protocols and life‐cycle assessments. Integrating these advances within a circular economy framework will accelerate the transition toward large‐scale, low‐carbon, and economically viable graphite regeneration.

Finally, integrating recycling processes into a circular economy framework, supported by standardized protocols, industrial collaboration, and comprehensive life cycle and economic assessments, will be essential to promote large‐scale, sustainable graphite regeneration. Future directions for spent graphite regeneration should focus on: 1) Developing eco‐friendly, high‐efficiency leaching systems using organic acids, salt solutions, or their combinations; 2) Exploring energy‐saving low‐temperature calcination and catalytic graphitization techniques; 3) Designing composite structures and low‐cost coatings to mitigate volume expansion and improve capacity retention; 4) Investigating the mechanisms of heteroatom doping and optimizing doping strategies to further enhance graphite performance. Together, these efforts will advance the sustainable recovery of graphite resources and enable the closed‐loop recycling of LIBs, contributing to the broader goals of carbon neutrality and resource circularity.

## Conflict of Interest

The authors declare no conflict of interest.

## References

[1] F. Arshad , L. Li , K. Amin , E. Fan , N. Manurkar , A. Ahmad , J. Yang , F. Wu , ACS. Sustain. Chem. Eng. 2020, 8, 13527.

[2] L. Zhao , L. Tian , J. Li , F. Shi , Y. Chang , J. Yan , H. Zhang , Energy Storage Mater. 2024, 71, 103640.

[3] S. C. Chelgani , M. Rudolph , R. Kratzsch , D. Sandmann , J. Gutzmer , Miner. Process. Extr. Metallurg. Rev. 2016, 37, 58.

[4] K. Zaghib , X. Song , A. Guerfi , R. Rioux , K. Kinoshita , J. Power Sources. 2003, 119, 8.

[5] S. Duehnen , J. Betz , M. Kolek , R. Schmuch , M. Winter , T. Placke , Small Methods 2020, 4, 2000039.

[6] D. Surovtseva , E. Crossin , R. Pell , L. Stamford , J. Ind. Ecol. 2022, 26, 964.

[7] K. Liu , S. Yang , L. Luo , Q. Pan , P. Zhang , Y. Huang , F. Zheng , H. Wang , Q. Li , Electrochim. Acta. 2020, 356, 136856.

[8] H. Wang , Y. Huang , C. Huang , X. Wang , K. Wang , H. Chen , S. Liu , Y. Wu , K. Xu , W. Li , Electrochim. Acta. 2019, 313, 423.

[9] H. Zhang , Y. Yang , D. Ren , L. Wang , X. He , Energy Storage Mater. 2021, 36, 147.

[10] S. Natarajan , M. L. Divya , V. Aravindan , J. Energy Chem. 2022, 71, 351.

[11] S. Natarajan , V. Aravindan , Adv. Energy Mater. 2020, 10, 2002238.

[12] X. Meng , Y. Xu , H. Cao , X. Lin , P. Ning , Y. Zhang , Y. G. Garcia , Z. Sun , Green Energy Environ. 2020, 5, 22.

[13] C. R. Birkl , M. R. Roberts , E. McTurk , P. G. Bruce , D. A. Howey , J. Power Sources. 2017, 341, 373.

[14] L. Kong , Y. Xing , M. G. Pecht , IEEE Access 2018, 6, 8387.

[15] R. Zhang , X. B. Cheng , C. Z. Zhao , H. J. Peng , J. L. Shi , J. Q. Huang , J. Wang , F. Wei , Q. Zhang , Adv. Mater. 2016, 28, 2155. 26754639 10.1002/adma.201504117

[16] X. Su , F. Dogan , J. Ilavsky , V. A. Maroni , D. J. Gosztola , W. Lu , Chem. Mater. 2017, 29, 6205.

[17] G. Wang , M. Yu , X. Feng , Chem. Soc. Rev. 2021, 50, 2388. 33346774 10.1039/d0cs00187b

[18] K. Takahashi , V. Srinivasan , J. Electrochem. Soc. 2015, 162, A635.

[19] N. Lin , Z. Jia , Z. Wang , H. Zhao , G. Ai , X. Song , Y. Bai , V. Battaglia , C. Sun , J. Qiao , J. Power Sources. 2017, 365, 235.

[20] C. Peng , L. Yang , S. Fang , J. Wang , Z. Zhang , K. Tachibana , Y. Yang , S. Zhao , J. Appl. Electrochem. 2010, 40, 653.

[21] S. Dai , J. Chen , Y. Ren , Z. Liu , J. Chen , C. Li , X. Zhang , X. Zhang , T. Zeng , Int. J. Electrochem. Sci. 2017, 12, 10589.

[22] J. Wojciechowski , Ł. Kolanowski , A. Bund , G. Lota , J. Power Sources. 2017, 368, 18.

[23] S. Ojanen , M. Lundström , A. Santasalo‐Aarnio , R. Serna‐Guerrero , Waste Manage. 2018, 76, 242. 10.1016/j.wasman.2018.03.045 29615279

[24] J. Shaw‐Stewart , A. Alvarez‐Reguera , A. Greszta , J. Marco , M. Masood , R. Sommerville , E. Kendrick , Sustain. Mater. Technol. 2019, 22, 00110.

[25] T. R. Grandjean , J. Groenewald , J. Marco , J. Energy Storage. 2019, 21, 202.

[26] L. P. Yao , Q. Zeng , T. Qi , J. Li , J. Clean. Prod. 2020, 245, 118820.

[27] Z. Shang , W. Yu , J. Zhou , X. Zhou , Z. Zeng , R. Tursun , X. Liu , S. Xu , ETrans. 2024, 20, 100320.

[28] J.‐R. Wang , D.‐H. Yang , Y.‐J. Xu , X.‐L. Hou , E. H. Ang , D.‐Z. Wang , L. Zhang , Z.‐D. Zhu , X.‐Y. Feng , X.‐H. Song , New Carbon Mater. 2023, 38, 787.

[29] J. C.‐Y. Jung , P.‐C. Sui , J. Zhang , J. Energy Storage. 2021, 35, 102217.

[30] S. B. Sep , Purif. Technol. 2017, 172, 388.

[31] X. Zhang , L. Li , E. Fan , Q. Xue , Y. Bian , F. Wu , R. Chen , Chem. Soc. Rev. 2018, 47, 7239. 30124695 10.1039/c8cs00297e

[32] L. Yun , D. Linh , L. Shui , X. Peng , A. Garg , M. L. P. Le , S. Asghari , S. J. Resour , Conserv. Recy. 2018, 136, 198.

[33] X.‐J. Nie , X.‐T. Xi , Y. Yang , Q.‐L. Ning , J.‐Z. Guo , M.‐Y. Wang , Z.‐Y. Gu , X.‐L. Wu , Electrochim. Acta. 2019, 320, 134626.

[34] S. Rothermel , M. Evertz , J. Kasnatscheew , X. Qi , M. Grützke , M. Winter , S. Nowak , ChemSusChem. 2016, 9, 3473. 27860314 10.1002/cssc.201601062

[35] J. Yang , E. Fan , J. Lin , F. Arshad , X. Zhang , H. Wang , F. Wu , R. Chen , L. Li , ACS Appl. Energy Mater. 2021, 4, 6261.

[36] C. Lin , A. Tang , H. Mu , W. Wang , C. Wang , J. Chem. 2015, 2015, 104673.

[37] Y. Bai , N. Muralidharan , J. Li , R. Essehli , I. Belharouak , ChemSusChem. 2020, 13, 5664.

[38] M. Ahuis , A. Aluzoun , M. Keppeler , S. Melzig , A. Kwade , J. Power Sources. 2024, 593, 233995.

[39] E. Trebeck , A. Grams , J. Talkenberger , S. Prakash , J. E. Grimmenstein , T. Krampitz , H. Lieberwirth , A. Valenas , Recycling 2025, 10, 189.

[40] L. De Vita , D. Callegari , A. Bianchi , C. Tealdi , N. Zucca , P. Galinetto , M. Colledani , E. Quartarone , ChemSusChem 2025, 18, 202500550. 10.1002/cssc.202500550 PMC12404021 40530751

[41] Q. He , C. Guo , K. Han , F. Liu , Y. Feng , X. Wang , X. Qian , J. Meng , Carbon 2025, 244, 120727.

[42] G. Wei , Y. Liu , B. Jiao , N. Chang , M. Wu , G. Liu , X. Lin , X. Weng , J. Chen , L. Zhang , Iscience 2023, 26, 107676. 37680490 10.1016/j.isci.2023.107676 PMC10480636

[43] Y. Gao , J. Zhang , H. Jin , G. Liang , L. Ma , Y. Chen , C. Wang , Carbon 2022, 189, 493.

[44] Z. Zhang , X. Zhu , H. Hou , L. Tang , J. Xiao , Q. Zhong , Waste Manage. 2022, 150, 30. 10.1016/j.wasman.2022.06.037 35792439

[45] H. Tian , M. Graczyk‐Zajac , D. M. De Carolis , C. Tian , E. I. Ricohermoso , Z. Yang , W. Li , M. Wilamowska‐Zawlocka , J. P. Hofmann , A. Weidenkaff , J. Hazard Mater. 2023, 445, 130607. 37056017 10.1016/j.jhazmat.2022.130607

[46] X. Ma , M. Chen , B. Chen , Z. Meng , Y. Wang , ACS Sustain. Chem. Eng. 2019, 7, 19732.

[47] J. Zhang , X. Li , D. Song , Y. Miao , J. Song , L. Zhang , J. Power Sources. 2018, 390, 38.

[48] B. Markey , M. Zhang , I. Robb , P. Xu , H. Gao , D. Zhang , J. Holoubek , D. Xia , Y. Zhao , J. Guo , J. Electrochem. Soc. 2020, 167, 160511.

[49] Z. Ma , Y. Zhuang , Y. Deng , X. Song , X. Zuo , X. Xiao , J. Nan , J. Power Sources. 2018, 376, 91.

[50] Y. Guo , F. Li , H. Zhu , G. Li , J. Huang , W. He , Waste Manage. 2016, 51, 227. 10.1016/j.wasman.2015.11.036 26674969

[51] Y. Yang , S. Song , S. Lei , W. Sun , H. Hou , F. Jiang , X. Ji , W. Zhao , Y. Hu , Waste Manage. 2019, 85, 529. 10.1016/j.wasman.2019.01.008 30803608

[52] Y. Xu , X. Song , Q. Chang , X. Hou , Y. Sun , X. Feng , X. Wang , M. Zhan , H. Xiang , Y. Yu , New Carbon Mater. 2022, 37, 1011.

[53] J. Li , Y. He , Y. Fu , W. Xie , Y. Feng , K. Alejandro , Waste Manage. 2021, 126, 517. 10.1016/j.wasman.2021.03.052 33839403

[54] S. Natarajan , R. M. Bhattarai , M. Sudhakaran , Y. S. Mok , S. J. Kim , J. Power Sources. 2023, 577, 233170.

[55] S. Ji , A. Zhang , W. Hua , S. Yan , X. Chen , Battery Energy 2024, 3, 20230067.

[56] Q. Chen , L. Huang , J. Liu , Y. Luo , Y. Chen , Carbon 2022, 189, 293.

[57] G. Zeng , R. Zhou , C. Hu , H. Zhao , H. Gao , J. Huang , J. Yu , F. Luo , Z. Wang , C. Deng , Carbon 2025, 238, 120182.

[58] Y. Gao , C. Wang , J. Zhang , Q. Jing , B. Ma , Y. Chen , W. Zhang , ACS. Sustain, Chem. Eng. 2020, 8, 9447.

[59] A. Chernyaev , A. Kobets , K. Liivand , F. Tesfaye , P.‐M. Hannula , T. Kallio , L. Hupa , M. Lundström , Miner. Eng. 2024, 208, 108587.

[60] Y. Li , W. Lv , H. Zhao , Y. Xie , D. Ruan , Z. Sun , Green Chem. 2022, 24, 9315.

[61] S. Natarajan , T. Mae , H. Y. Teah , H. Sakurai , S. Noda , J. Mater. Chem A. 2025, 13, 4984.

[62] K. E. Haque , Int. J. Miner Process. 1999, 57, 1.

[63] W. Fan , J. Zhang , R. Ma , Y. Chen , C. Wang , J. Electroanal. Chem. 2022, 908, 116087.

[64] D. Hou , Z. Guo , Y. Wang , X. Hou , S. Yi , Z. Zhang , S. Hao , D. Chen , Surf. Interfaces 2021, 24, 101098.

[65] J. Jegan Roy , E. J. J. Tang , M. P. Do , B. Cao , M. Srinivasan , ACS. Sustain. Chem. Eng. 2023, 11, 6567.

[66] S.‐H. Zheng , X.‐T. Wang , Z.‐Y. Gu , H.‐Y. Lü , S. Li , X.‐Y. Zhang , J.‐M. Cao , J.‐Z. Guo , X.‐L. Wu , J. Colloid. Interface Sci. 2024, 667, 111. 38626654 10.1016/j.jcis.2024.04.058

[67] J. Xiong , Y. Wang , J. Lu , F. Xi , Z. Tong , W. Ma , S. Li , J. Colloid. Interface Sci. 2025, 685, 555. 39855096 10.1016/j.jcis.2025.01.175

[68] T. Li , L. Tao , L. Xu , T. Meng , B. C. Clifford , S. Li , X. Zhao , J. Rao , F. Lin , L. Hu , Adv. Funct. Mater. 2023, 33, 2302951.

[69] Y. Ji , H. Zhang , D. Yang , Y. Pan , Z. Zhu , X. Qi , X. Pi , W. Du , Z. Cheng , Y. Yao , Adv. Mater. 2024, 36, 2312548. 10.1002/adma.202312548 38323869

[70] W. Chen , R. V. Salvatierra , J. T. Li , C. Kittrell , J. L. Beckham , K. M. Wyss , N. La , P. E. Savas , C. Ge , P. A. Advincula , Adv. Mater. 2023, 35, 2207303. 10.1002/adma.202207303 36462512

[71] J. Luo , J. Zhang , Z. Guo , Z. Liu , S. Dou , W.‐D. Liu , Y. Chen , W. Hu , Nano Res. 2023, 16, 4240.

[72] Z. Shang , Z. Naizhe , Z. Ying , D. Zou , F. Dai , J. Xie , J. Shao , X. Liu , S. Xu , Chem. Eng. J. 2025, 505, 159132.

[73] X. Li , B. Wu , H. Sun , K. Zhu , Y. Gao , T. Bao , H. Wu , D. Srinivasan , Sus. Energy Fuels 2024, 8, 1438.

[74] K. Liivand , M. Kazemi , P. Walke , V. Mikli , M. Uibu , D. D. Macdonald , I. Kruusenberg , ChemSusChem. 2021, 14, 1103. 33314598 10.1002/cssc.202002742

[75] D. Ruan , K. Zou , K. Du , F. Wang , L. Wu , Z. Zhang , X. Wu , G. Hu , ChemCatChem. 2021, 13, 2025.

[76] B. Wei , S. Yang , Chem. Eng. J. 2021, 404, 126437.

[77] P. Wang , J. Zhang , L. Dong , C. Sun , X. Zhao , Y. Ruan , H. Lu , Chem. Mater. 2017, 29, 3412.

[78] E. G. da Silveira Firmiano , A. C. Rabelo , C. J. Dalmaschio , A. N. Pinheiro , E. C. Pereira , W. H. Schreiner , E. R. Leite , Adv. Energy Mater. 2014, 4, 1301380.

[79] Y. Zhao , H. Wang , X. Li , X. Yuan , L. Jiang , X. Chen , J. Hazard Mater. 2021, 420, 126552. 34329073 10.1016/j.jhazmat.2021.126552

[80] Y. Wang , H. Cao , L. Chen , C. Chen , X. Duan , Y. Xie , W. Song , H. Sun , S. Wang , Appl. Catal B: Environ. 2018, 229, 71.

[81] Y. Zhang , N. Song , J. He , R. Chen , X. Li , Nano Lett. 2018, 19, 512. 30567438 10.1021/acs.nanolett.8b04410

[82] S. Natarajan , H. C. Bajaj , V. Aravindan , J. Mater. Chem A. 2019, 7, 3244.

[83] M. Divya , S. Natarajan , Y.‐S. Lee , V. Aravindan , J. Mater. Chem A. 2020, 8, 4950.

[84] L. Yang , L. Yang , G. Xu , Q. Feng , Y. Li , E. Zhao , J. Ma , S. Fan , X. Xi , Sci. Rep. 2019, 9, 9823. 31285508 10.1038/s41598-019-46393-4 PMC6614457

[85] S. Natarajan , D. S. Lakshmi , H. C. Bajaj , D. N. Srivastava , J. Environ. Chem. Eng. 2015, 3, 2538.

[86] W. Chen , H. Qu , R. Shi , J. Wang , H. Ji , Z. Zhuang , J. Ma , D. Tang , J. Li , J. Tang , ACS Energy Lett. 2024, 9, 3505.

[87] H. Da , S. Pan , J. Li , J. Huang , X. Yuan , H. Dong , J. Liu , H. Zhang , Energy Storage Mater. 2023, 56, 457.

[88] Z. Cheng , Z. Luo , H. Zhang , W. Zhang , W. Gao , Y. Zhang , L. Qie , Y. Yao , Y. Huang , K. K. Fu , Carbon Energy 2024, 6, 395.

[89] S. Tu , B. Zhang , Y. Zhang , Z. Chen , X. Wang , R. Zhan , Y. Ou , W. Wang , X. Liu , X. Duan , Nat. Energy 2023, 8, 1365.

[90] C. Wang , Y. Xie , Y. Huang , S. Zhou , H. Xie , H. Jin , H. Ji , Angew. Chem., Int. Ed. 2024, 63, 202402301. 10.1002/anie.202402301 38482741

[91] Y. Hou , H. Guo , B. Xing , H. Zeng , W. Kang , X. Qu , C. Zhang , J. Jia , G. Huang , Y. Cao , Fuel 2024, 374, 132488.

[92] D. Ruan , F. Wang , L. Wu , K. Du , Z. Zhang , K. Zou , X. Wu , G. Hu , New J. Chem. 2021, 45, 1535.

[93] S. Luo , F. Liu , W. Tianxu , Y. Liu , C. Zhang , C. Bie , M. Liu , P. K. Chu , K. Huo , B. Gao , Energy Storage Mater. 2024, 73, 103833.

[94] B. Wang , Y. Dong , Z. Zeng , H. Lei , Y. Yang , P. Ge , J. Power Sources. 2024, 621, 235299.

[95] Y. Gao , J. Zhang , Y. Chen , C. Wang , Surf. Interfaces. 2021, 24, 101089.

[96] Z. Li , S. Li , T. Wang , K. Yang , Y. Zhou , Z. Tian , J. Electrochem. Soc. 2021, 168, 090513.

[97] H. Da , M. Gan , D. Jiang , C. Xing , Z. Zhang , L. Fei , Y. Cai , H. Zhang , S. Zhang , ACS. Sustain. Chem. Eng. 2021, 9, 16192.

[98] M. Baek , J. Kim , J. Jin , J. W. Choi , Nat. Commun. 2021, 12, 6807. 34815396 10.1038/s41467-021-27095-w PMC8611023

[99] E. Kazyak , K. H. Chen , Y. Chen , T. H. Cho , N. P. Dasgupta , Adv. Energy Mater. 2022, 12, 2102618.

[100] J. Zhou , K. Ma , X. Lian , Q. Shi , J. Wang , Z. Chen , L. Guo , Y. Liu , A. Bachmatiuk , J. Sun , Small 2022, 18, 2107460. 10.1002/smll.202107460 35224838

[101] J. Seo , S. Hyun , J. Moon , J. Y. Lee , C. Kim , ACS Appl. Energy Mater. 2022, 5, 5610.

[102] H.‐Y. Wang , F.‐M. Wang , J. Power sources. 2013, 233, 1.

[103] F. Zou , H. C. Nallan , A. Dolocan , Q. Xie , J. Li , B. M. Coffey , J. G. Ekerdt , A. Manthiram , Energy Storage Mater. 2021, 43, 499.

[104] L. Wang , Y. Zhao , J. Sun , Y. Li , Q. Qu , H. Zheng , Carbon 2024, 230, 119656.

[105] K. Cai , C. Xiang , X. Wang , X. Zhang , D. Zhang , Z. Zheng , H. Jin , X. Li , L. Li , J. Electrochem. Soc. 2024, 171, 030505.

[106] C. Xu , G. Ma , W. Yang , S. Che , Y. Li , Y. Jia , H. Liu , F. Chen , G. Zhang , H. Liu , Electrochim. Acta. 2022, 415, 140198.

[107] T. Deng , X. Zhou , Mater. Lett. 2016, 176, 151.

[108] J. Xu , X. Wang , B. Hu , J. Ding , Z. Zhang , S. Ge , Batteries Supercaps 2023, 6, 202200499.

[109] Q. Cheng , R. Yuge , K. Nakahara , N. Tamura , S. Miyamoto , J. Power Sources. 2015, 284, 258.

[110] X. Yang , H. Zhen , H. Liu , C. Chen , Y. Zhong , X. Yang , X. Wang , L. Yang , Waste Manage. 2023, 161, 52. 10.1016/j.wasman.2023.02.032 36863210

[111] X. Zhu , J. Xiao , Y. Chen , L. Tang , H. Hou , Z. Yao , Z. Zhang , Q. Zhong , Chem. Eng. J. 2022, 450, 138113.

[112] S. Chae , S. H. Choi , N. Kim , J. Sung , J. Cho , Angew. Chem., Int. Ed. 2020, 59, 110. 10.1002/anie.201902085 30887635

[113] M. Ko , S. Chae , J. Ma , N. Kim , H.‐W. Lee , Y. Cui , J. Cho , Nat. Energy 2016, 1, 16113.

[114] A. Bai , L. Wang , J. Li , X. He , J. Wang , J. Wang , J. Power Sources. 2015, 289, 100.

[115] H. Jin , S. Xin , C. Chuang , W. Li , H. Wang , J. Zhu , H. Xie , T. Zhang , Y. Wan , Z. Qi , Science 2020, 370, 192. 33033214 10.1126/science.aav5842

[116] X. Xiao , X. Li , S. Zheng , J. Shao , H. Xue , H. Pang , Adv. Mater. Interfaces. 2017, 4, 1600798.

[117] H. Ying , W. Q. Han , Adv. Sci. 2017, 4, 1700298. 10.1002/advs.201700298 PMC5700643 29201624

[118] D. Ruan , L. Wu , F. Wang , K. Du , Z. Zhang , K. Zou , X. Wu , G. Hu , J. Electroanal. Chem. 2021, 884, 115073.

